# A diet-induced obese and diabetic host phenotype reduces mosquito ZIKV infections and remodels gut metabolism

**DOI:** 10.3389/fimmu.2025.1704301

**Published:** 2025-12-15

**Authors:** Alexandre Menezes, Ana Beatriz Walter-Nuno, Emylle Costa-Bartuli, Daniel Moreira, Tatiana El-Bacha, Ana Paula Méndez, Anderson Amarante, Nathan Kistenmacker, Pâmela Huaman, Mileane Busch, Jéssica Pereira, Isabela Ramos, Georgia Atella, Thiago Parente, Gabriela Paiva-Silva, Kildare Miranda, Patricia Zancan, Mauro Sola-Penna, Fabio M. Gomes

**Affiliations:** 1 Laboratório de Ultraestrutura Celular Hertha Meyer, Universidade Federal do Rio de Janeiro, Rio de Janeiro, Brazil; 2 Laboratório de Bioquímica e Biologia Molecular de Artrópodes Hematófagos, Universidade Federal do Rio de Janeiro, Rio de Janeiro, Brazil; 3 Instituto Nacional de Ciência e Tecnologia em Entomologia Molecular, Rio de Janeiro, Brazil; 4 The metaboliZSm’ grouP (ZSP group), Universidade Federal do Rio de Janeiro, Rio de Janeiro, Brazil; 5 Laboratório de Genômica Aplicada e Bioinovações, Fundação Oswaldo Cruz, Rio de Janeiro, Brazil; 6 LeBioME-Bioactives, Universidade Federal do Rio de Janeiro, Rio de Janeiro, Brazil; 7 Laboratório de Bioquímica de Lipídeos e Lipoproteínas, Universidade Federal do Rio de Janeiro, Rio de Janeiro, Brazil; 8 Laboratorio de Ovogênese Molecular de Insetos Vetores, Universidade Federal do Rio de Janeiro, Rio de Janeiro, Brazil

**Keywords:** mosquito, *Aedes*, Zika (ZIKV), arbovirus, obesity, diabetes, immunometabolism, diet

## Abstract

**Introduction:**

Arbovirus infections, including dengue, Zika, and chikungunya, constitute significant global health threats. The epidemiology of these diseases is closely tied to the biology and ecology of the mosquito *Aedes aegypti*, particularly regarding its vector competence—the mosquito’s ability to acquire, maintain, and transmit pathogens. While genetic variations among mosquito populations have traditionally received the most attention and are often regarded as the main determinants of vector competence, life history components, including immune history, microbiota composition, and nutritional status, are increasingly recognized as critical modulators of this trait. In this context, the increasing prevalence of diet-induced obesity and diabetes in human hosts—a condition that alters blood plasma composition—may reshape the mosquito´s nutritional and immunological landscape.

**Methods:**

This study investigated the impact of these conditions on *A. aegypti* biology and Zika virus (ZIKV) infection. For this, AG129 mice were fed a high-fat, high-sucrose (HFHS) diet for 20 weeks to develop weight gain and insulin resistance. By comparing mosquitoes fed on healthy and diabetic-obese mice, we assessed changes in life history traits, immunometabolic parameters, and transcriptomic profiles.

**Results:**

Notably, mosquitoes fed on HFHS-fed mice showed reduced survival, altered lipid profile and a significant reduction in midgut and systemic ZIKV infection levels, which correlated with distinct transcriptomic alterations in genes related to gut metabolism and homeostasis.

**Discussion:**

These findings demonstrate that the host’s metabolic state is a critical modulator of mosquito physiology, increasing mosquito mortality while reducing ZIKV infection levels. This highlights that host-centric factors, such as the rising prevalence of metabolic syndrome, are an overlooked variable that may have complex epidemiological consequences for arbovirus transmission by mosquitoes.

## 1 Introduction

Arthropod-borne viruses (arboviruses) transmitted by *Aedes* mosquitoes, such as dengue, Zika, and chikungunya, pose significant global health threats due to their rapid spread and potential to cause severe diseases (1, 2). Key factors such as vector competence—the intrinsic ability of mosquitoes to acquire, sustain, and transmit pathogens—and vectorial capacity—the combination of vector competence, as well as ecological and behavioral attributes that determine the efficiency of disease transmission —play critical roles in shaping the spread and impact of these infections (3, 4). These factors are further intensified by climate change, which facilitates the expansion of mosquito habitats, accelerates mosquito developmental cycles, and creates optimal breeding environments for the expansion of mosquito populations (5). Altogether, they enhance the transmission dynamics of mosquito-borne diseases and have led to record numbers of cases in the Americas (6).

Variations in vector competence among different natural populations of *Aedes aegypti* are well documented (7), and previous genetic studies have mapped genetic loci associated with susceptibility to infections in mosquitoes and other insects (8, 9). However, vector competence is not solely determined by the mosquito’s genetic background; it is also influenced by individual life history and events that modulate mosquito physiology and immunity (3, 4, 10–12). For instance, prior exposure to pathogens or immune stimuli during larval and adult stages can prime mosquitoes´ immune systems and alter their interactions with pathogens (13). Moreover, the composition of the mosquito microbiota, which is tightly linked to their habitat (14, 15), plays a crucial role in shaping immune responses during infections (16, 17). Nutrition, particularly the composition of blood meals, also seems to impact mosquito immunity by regulating vector immunometabolism (18–20). Various blood components, including macronutrients (21, 22), micronutrients (23), hormones (24), and cytokines (25), have been shown to influence metabolic pathways that intersect with immune signaling networks (26, 27). Consequently, physiological alterations in host blood composition, driven by diet and metabolic diseases, are emerging as significant modulators of mosquito vector biology (28, 29).

Hosts consuming Western diets, characterized by excessive intake of ultraprocessed foods, exhibit marked alterations in blood plasma composition, including elevated content of lipids, glucose, hormones, and inflammatory cytokines (30). This situation becomes critical as the prevalence of metabolic syndrome has reached pandemic levels, mostly as a result of the widespread consumption of Western diets, and is likely to increase (31, 32). More than 1 billion people worldwide now live with obesity, with rates having doubled among adults and quadrupled among children since 1990 (33). Similarly, more than 5% of the world population lived with type 2 diabetes in 2019, with a 1.5% annual increase in prevalence (34).

In a previous study, we demonstrated that *A. aegypti* mosquitoes feeding on mice subjected to dietary protocols mimicking a Western Diet exhibited altered vector capacity, including reduced longevity (28). Here, we further explore the impact of host diet-induced obesity and diabetes on mosquito vector biology and on the transcriptomic profile and Zika virus (ZIKV) infections in the mosquito midgut - the primary barrier to arbovirus dissemination following blood feeding (35, 36). Notably, we observed a reduction in ZIKV infection levels in mosquitoes that fed on the blood of obese and diabetic mice, which correlated with distinct physiological and metabolic alterations identified through RNA-Seq analysis. These findings have implications for public health strategies to control ZIKV and other arbovirus-borne diseases, particularly in populations undergoing nutritional transitions toward Westernized diets, as seen in many low and middle-income countries (LMICs).

## 2 Materials and methods

### 2.1 Mice dietary intervention

Animals were housed in the (NBA2) Rodent Experimental Animal Facility at IBCCF/UFRJ, maintained in individually ventilated racks (IVCs) (Alesco, Brazil) within rooms featuring controlled environmental conditions (21 ± 1°C, 55% humidity, 15 macro- and micro-environmental air changes per hour). All components of the mini-isolators (cages, grates, lids, and water bottles), as well as the standardized chow (NUVILAB, Brazil), filtered water, and pine bedding, were sterilized by moist heat in autoclaves prior to use. The efficacy of this sterilization process is routinely certified by biological and chemical indicator tests. All procedures followed the guidelines of the National Council for the Control of Animal Experimentation (CONCEA) and the NIH Guide for the Care and Use of Laboratory Animals. Animal health and environmental parameters were continuously monitored by a specialized in-house technical team and experimental procedures involving animals were conducted according to the ethical guidelines approved by the Animal Care and Use Committee of the Health Sciences Center, Federal University of Rio de Janeiro (protocol number CEUA/CCS/UFRJ 087/20). Briefly, six-week-old AG129 mice were randomly divided into two dietary groups. The control group received a standard CHOW AIN93M diet, while the experimental group received a high-fat, high-sucrose (HFHS) diet designed to induce overweight and insulin resistance when compared to CHOW controls (28). The specific compositions of the CHOW and HFHS diets are detailed in the supplemental files (**Supplementary Table 1**). The body weights of all mice were measured weekly using a calibrated digital balance to monitor weight gain and confirm the development of overweight.

To assess metabolic health, insulin tolerance tests (ITT) and oral glucose tolerance tests (oGTT) were conducted at the 19th and 20th weeks of the dietary protocol, as described (28). For the oral glucose tolerance test (oGTT), mice were fasted for 5 hours and given an oral dextrose dose of 2 g/kg body weight. Blood glucose levels were measured from tail vein blood samples at baseline (0 minutes) and at 15, 30, 60, and 120 minutes post-administration using a FreeStyle Precision Neo glucometer (Abbott Laboratories, Chicago, IL, USA). The insulin tolerance test (ITT) was performed in the 19th week to evaluate insulin resistance. Mice were fasted for 5 hours before receiving an intraperitoneal insulin injection at 0.5 U/kg body weight. Blood samples were collected from the tail vein immediately before the injection and at 15, 30, 60, and 120 minutes post-injection to measure blood glucose levels using the glucometer. For blood metabolic measurement, after the 20-week dietary intervention, mice were immobilized and anesthetized (100mg/kg ketamine, 10 mg/kg xylazine), and blood was collected by cardiac puncture. Following cardiac puncture, mice were euthanized by cervical dislocation while still under anesthesia. Serum was then separated by gentle centrifugation (300g, 10 min, 4°C) and stored at -80°C. Insulin was measured by an enzyme-linked immunosorbent assay (ELISA) with the Insulin Mouse ELISA Kit (ThermoFisher, Carlsbad, CA).

### 2.2 NMR-based metabolomic analysis of mouse serum

For NMR analysis, serum samples were quickly thawed and diluted 3-fold in sodium phosphate buffer and deuterium oxide (50 mM phosphate buffer and 10% deuterium oxide, pH 7.4). A total of 600 μL of diluted samples was transferred to a 5 mm NMR tube. NMR spectra were acquired on a Bruker Advance III at 500 MHz at 298 K, coupled with a cooled automatic sample case at 280 K, and the software ICON NMR (Bruker) was used for automatic acquisition. 1D-1H NMR spectra were acquired using excitation sculpting to suppress the water signal (37), as well as a CPMG (Carr-Purcell-Meiboom-Gill) T2 filter (38) with 32 loop counters and a delay of 0.001 s. 32,768 complex data points were acquired per transient for a total of 1,024 transients. The spectral width was set to 19.99 ppm, resulting in an acquisition time of 3.27 s per free induction decay (FID). The relaxation delay was set to 1.74 s.

Spectra were pre-processed using the MetaboLabPy software (39). Before the Fourier transform, the FIDs were apodized using an exponential window function with 0.3 Hz line-broadening and then zero-filled to 65,536 real data points. After the Fourier transform, NMR spectra were manually phased, baseline corrected, referenced to the α-glucose anomeric doublet at δ 5.223 ppm, and aligned using the Icoshift algorithm (40), which is integrated into the MetabolabPy software. Regions corresponding to water and ethanol signals were excluded. Additionally, noise filtering was performed, and anything below 5 times the noise threshold, measured between 9.5 and 10 ppm, was discarded. All spectra were normalized using probabilistic quotient normalization (41) to reduce the effects of sample dilution or concentration of the compounds. Spectra were exported to the Chenomx NMR Suite 9.0^®^ program (Chenomx Inc.) for metabolite assignment and quantification.

### 2.3 Mosquito husbandry

*A. aegypti* mosquitoes (Red Eye strain) were reared under controlled laboratory conditions at the insectary (27 +/- 1°C, 70% relative humidity with a 12:12h light:dark cycle). Larvae were maintained in 1 L of filtered water and fed 1.5 g of Pedigree dog chow at the density of 100 larvae/L. Pupae were collected and transferred to a 3L plastic cage for adult emergence, and maintained at the same temperature and humidity conditions. Adult mosquitoes were given a 10% sucrose solution and allowed to mate before being used in experiments. To assess the impact of altered blood composition from obese and diabetic mice on mosquito biology, groups of 50 female mosquitoes, aged 5 to 6 days, were allowed to feed directly on anesthetized AG129 mice. Mice were anesthetized using a combination of 10 mg/kg xylazine and 100 mg/kg ketamine to facilitate feeding. Sugar sources were removed from the mosquito environment the night before feeding to induce higher feeding rates. Mosquitoes were allowed to feed for 30 minutes, after which unfed or partially engorged mosquitoes were removed from the cages. At the conclusion of the feeding period, mice were euthanized by cervical dislocation while still under anesthesia.

### 2.4 Survival and oviposition analysis

*A. aegypti* mosquitoes, aged 5 to 6 days, were allowed to feed on anesthetized AG129 mice. Following the blood meal, mosquitoes were maintained under standard laboratory conditions. *A. aegypti* mosquitoes were monitored daily for survival following a blood meal from AG129 mice. Post-feeding, unfed mosquitoes were removed, and the remaining mosquitoes were maintained under standard laboratory conditions. Survival was tracked daily for 10 days to track early mortality as a result of blood ingestion. Individual mosquito data was pooled from independent experiments and analyzed to generate survival curves. Oviposition was assessed by allowing mosquitoes to lay eggs individually. Two days post-blood meal (dpbm), individual mosquitoes were placed in small oviposition chambers containing moistened filter paper as a substrate for egg laying. Mosquitoes were allowed to lay eggs for over 48 hours. Eggs laid by each mosquito were manually counted using a stereomicroscope. The total number of eggs laid was recorded for each mosquito, and individual mosquito data was pooled from independent experiments to compare oviposition rates between the dietary groups.

### 2.5 Fat body lipid labeling and quantification

*A. aegypti* mosquitoes, aged 5 to 6 days, were allowed to feed on anesthetized AG129 mice. Following the blood meal, mosquitoes were maintained under standard laboratory conditions. Mosquitoes were collected and dissected at two time points: 2 and 4 dpbm. Dissections were performed on cold-anesthetized mosquitoes to isolate the fat body tissues. Tissues were incubated in a solution of 5 nM BODIPY 493/503 (Thermo Fisher Scientific) diluted in PBS for 30 minutes at room temperature, protected from light. Following incubation, tissues were washed three times with PBS to remove excess dye. The fat body tissues were incubated with 10 µg/ml DAPI (Thermo Fisher Scientific) for nuclei labeling. The whole tissue was mounted on glass slides with ProLong Gold Antifade Mountant (Thermo Fisher Scientific) and imaged using a Zeiss Elyra laser scanning microscope equipped with an EC Plan-Neofluar 10x/0.30 M27 objective. To capture the entire fat body, 25-tile scans were acquired using a LineSequential scan mode. We used a 488 nm laser to excite BODIPY (emission collected at 493–589 nm) and a 405 nm laser to excite DAPI (emission collected at 410–483 nm). All other acquisition parameters, such as detector gain, laser power, and pinhole settings, were held constant for all samples to ensure consistent and comparable intensity measurements. Tile scans were acquired to visualize the entire dissected fat body tissue. A region of interest (ROI) encompassing the entire tissue was manually drawn for each sample, and the mean fluorescence intensity of a single tissue was measured using ZEN software. To account for variations in tissue size, fluorescence intensity data were normalized to the total area of the selected tissue. Autofluorescence was assessed using fat bodies processed in parallel without the BODIPY 493/503 dye; at the acquisition settings used for imaging, background fluorescence was negligible.

### 2.6 Gas chromatography–mass spectrometry fatty acid profiling

*A. aegypti* mosquitoes, aged 5 to 6 days, were allowed to feed on anesthetized AG129 mice. Following the blood meal, mosquitoes were maintained under standard laboratory conditions. Dissections were performed on cold-anesthetized mosquitoes to isolate the fat body tissues at two time points (2 dpbm and 4 dpbm), and stored at -80°C in batches containing 15 tissues each. Following, dissected tissues were dissolved in 1 mL of toluene in a glass tube. Subsequently, 2 mL of 1% sulfuric acid in methanol was added to the solution. The mixture was incubated overnight at 50°C in a stoppered tube to facilitate esterification. Following incubation, 1 mL of a 5% sodium chloride aqueous solution was added to the mixture. The fatty acid methyl esters (FAMEs) were extracted using hexane (2 x 2 mL). Phase separation was achieved using Pasteur pipettes. The hexane layer containing the FAMEs was evaporated under a gentle stream of nitrogen gas. The dried FAMEs were then reconstituted in 100 µL of heptane. Methyl nonadecanoate (Sigma-Aldrich) served as the internal standard. GC-MS analysis was performed on a Shimadzu GCMS-QP2010 Plus system equipped with an HP Ultra 2 column (5% phenyl-methylpolysiloxane, Agilent; 25 m length x 0.20 mm inner diameter x 0.33 µm film thickness). The injector temperature was set at 250°C. The column temperature program was as follows: an initial hold at 40°C, ramped to 160°C at a rate of 30°C/min, then to 233°C at 1°C/min, and finally to 300°C at 30°C/min with a hold for 10 minutes. Helium served as the carrier gas with a linear velocity of 36.0 cm/s. A sample volume of 1 µL was injected into the chromatograph. Electron ionization (EI) was conducted at 70 eV, and mass analysis was performed using a quadrupole mass analyzer scanning from 40 to 440 amu. Both the interface and ion source temperatures were maintained at 240°C. Component identification was achieved by comparing mass spectra against the NIST05 library within the mass spectrometer software. Retention indices were further used to confirm peak identities by comparison with the Supelco 37 Component FAME Mix standard curves using 9:0 and 19:0 methyl esters (SIGMA-Aldrich) as internal standards.

### 2.7 Zika virus infection and quantification of viremia

Standard membrane-feeding techniques were employed for ZIKV infection assays to ensure that mosquitoes were exposed to the same viral titer during infection. For that, CHOW or HFHS mice were anesthetized using 10 mg/kg xylazine and 100 mg/kg ketamine, blood was collected intraperitoneally, and ZIKV was added to the blood mixture to guarantee equal concentration of virus load in each condition. Mosquitoes were fed blood meals containing 10^6^ pfu ZIKV [ZIKV strain ZIKV/H.sapiens/Brazil/PE243/201 (GenBank accession number KX197192.1)] using an artificial membrane feeder system for 30 min, and blood was kept at 37°C using a water heater. Infection rates were determined by dissecting midguts from cold-anesthetized mosquitoes at 4 and 8 dpbm. Individual midguts from cold-anesthetized mosquitoes were dissected, temporarily stored at dry ice over during the period of batch dissection, and later stored at -80°C. RNA was extracted from individual midguts using Trizol reagent (Invitrogen), and cDNA was synthesized using standard protocols from High-Capacity cDNA Reverse Transcription Kit (Applied Biosystems) for virus quantification. Quantitative reverse transcription PCR (qRT-PCR) was performed using PowerUp Sybr (Thermo Fisher Scientific) to quantify viral RNA levels using previously validated PAHO primers (FOR: CTGTGGCATGAACCCAATAG; REV: ATCCCATAGAGCACCACTCC) (42), normalizing against the expression of the housekeeping gene rp49 AAEL003396 (FOR: GCTATGACAAGCTTGCCCCCA; REV: TCATCAGCACCTCCAGCT) (28). Individual mosquito data was pooled from independent experiments to compare infection levels between CHOW and HFHS-fed mosquitoes.

### 2.8 RNA-seq analysis of gene expression

Standard membrane-feeding techniques were employed for ZIKV infection assays. CHOW or HFHS mice were anesthetized using 10 mg/kg xylazine and 100 mg/kg ketamine, blood was collected intraperitoneally, and ZIKV was added to the blood mixture to guarantee equal concentration of virus load in each condition. Mosquitoes were fed blood meals containing 10^6^ pfu ZIKV [ZIKV strain ZIKV/H.sapiens/Brazil/PE243/201 (GenBank accession number KX197192.1)] using an artificial membrane feeder system for 30 min, and blood was kept at 37°C using a water heater. A parallel batch of uninfected mosquitoes was prepared by allowing mosquitoes to feed from the blood of CHOW or HFHS mice without the addition of ZIKV. Midguts from cold-anesthetized mosquitoes were dissected at 1 and 4 dpbm, temporarily stored at dry ice over during the period of batch dissection, and later stored at -80°C. Following, total RNA was extracted from 15 midguts per independent groups using Trizol reagent (Invitrogen). The assessment of RNA integrity, library preparation, and sequencing was performed at the NGS Sequencing Facility at Fiocruz, Rio de Janeiro. RNA integrity was evaluated using TapeStation (Agilent). Only samples with a RIN above 7.0 were used for library preparation, utilizing the Illumina Stranded mRNA kit with Illumina RNA UD Indexes Set A (Illumina), strictly following the provided protocols. Library sequencing from 4 independent replicates per condition was performed on a NextSeq2000 (Illumina) using a P4 XLEAP-SBS cartridge, generating approximately 18 million 50bp single-end reads per sample. The raw sequencing reads were made available through the NCBI Sequence Read Archive (SRA) via the BioProject accession number PRJNA1291278.

After sequencing, the obtained data underwent a quality assessment using FastQC v0.11.9 (https://www.bioinformatics.babraham.ac.uk/projects/fastqc/). Subsequently, the software STAR v2.7.5c (43) was employed for mapping and quantifying the number of reads that uniquely aligned to each gene of the *A. aegypti* genome (AaegL5). Differential expression analysis was carried out using the R package DESeq2 (parameters: p-adj < 0.05 and |LFC| > 0) (44), while Gene Set Enrichment Analysis (GSEA) was performed using ClusterProfiler (45) with the KEGG pathway as the reference database.

## 3 Results

### 3.1 A Western diet protocol induces overweight and insulin resistance in AG129 mice

**Figure 1:**
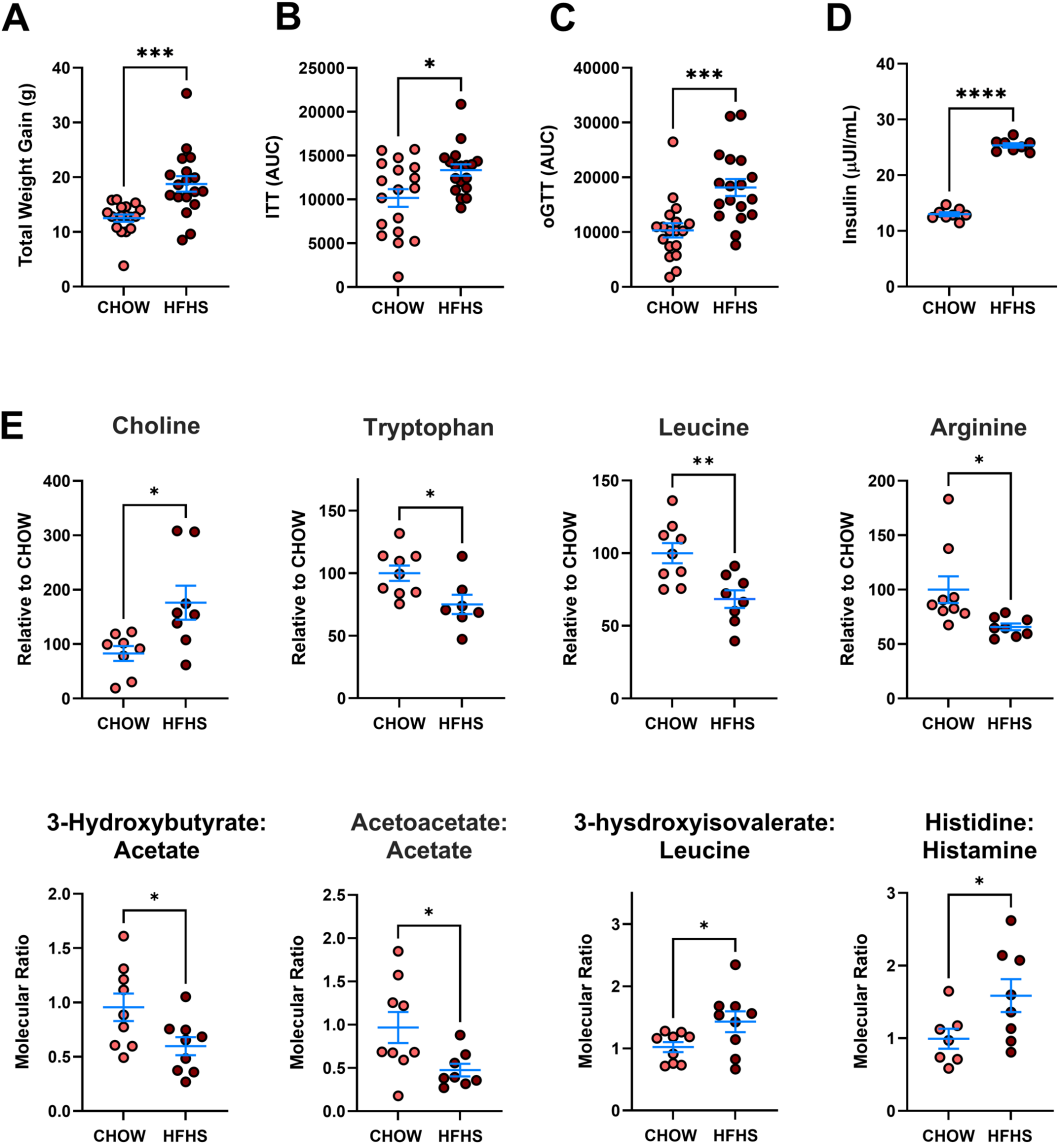
A 20-week HFHS diet induces an obese, insulin-resistant phenotype and a distinct serum metabolomic profile in AG129 mice. Mice were either fed a high-fat, high-sucrose (HFHS) diet or a standard chow (CHOW) diet for 20 weeks. **(A)** Over the period, mice were weighed weekly, and the total weight gain is depicted. **(B)** At the 19th week of dietary protocol, insulin sensitivity was evaluated by the Insulin Tolerance Test (ITT), indicating the response to exogenous insulin **(C)**. In the 20th week, glucose tolerance was assessed by the Oral Glucose Tolerance Test (oGTT), measuring glucose clearance post-administration. After mice euthanasia, serum was collected, and **(D)** the resting insulin levels were measured. **(E)** Mice serum metabolomic profile was analyzed via Nuclear Magnetic Resonance (NMR) analysis. The relative concentration of selected metabolites or the molecular ratio between metabolites is shown. Bars represent Mean +/- SEM. Statistical significance was determined using the unpaired t-test *P<0.05, **P<0.01, ***P<0.001, ****P<0.0001. A total of **(A–C)**: 18 mice per group; **(D)** 8 mice per group; **(E)** and at least 7 mice per group were used.

AG129 mice were fed either a previously validated high-fat, high-sugar (HFHS) diet or a standard chow (CHOW) diet for 20 weeks to induce overweight and insulin resistance (46) (**Figure 1**; **Supplementary Table 2**). The HFHS diet significantly increased total body weight gain over the 20 weeks compared to the CHOW diet (CHOW: 12.53 ± 2.82 g, HFHS: 18.76 ± 6.04 g, P < 0.001) (**Figure 1A**). Insulin and glucose tolerance tests showed markedly impaired glucose homeostasis in HFHS-fed mice. The ITT conducted at week 19 revealed that CHOW-fed mice had significantly better insulin sensitivity than HFHS-fed mice (CHOW: 10.169 ± 4.264 AU, HFHS: 13.349 ± 2.763 AU, P < 0.05) (**Figure 1B**). Similarly, the oGTT showed CHOW-fed mice cleared glucose more effectively than the HFHS group (CHOW: 13.313 ± 5.520 AU, HFHS: 18.139 ± 6.585 AU, P < 0.001) (**Figure 1C**). After 20 weeks of dietary intervention, mice were euthanized, and blood serum was collected and analyzed. ELISA assays revealed significantly lower insulin levels in CHOW-fed controls compared to HFHS-fed mice (CHOW: 13.01 ± 1.00 μUI/mL, HFHS: 25.34 ± 1.07 μUI/mL, P < 0.0001) (**Figure 1D**).

Nuclear Magnetic Resonance (NMR)-based metabolomic profiling further demonstrated distinct changes in serum metabolite concentrations (**Figure 1E**). Specifically, HFHS-fed mice showed a significant increase in choline (P < 0.05) levels, and decreased levels in circulating amino acids, particularly tryptophan (P < 0.05), leucine (P < 0.01), and arginine (P < 0.05). To further explore pathway activity, molecular ratios of selected metabolite pairs were calculated. Ratios related to ketone body metabolism were reduced in HFHS mice, including 3-hydroxybutyrate to acetate (P < 0.05) and acetoacetate to acetate (P < 0.05). Ratios reflecting branched-chain amino acid catabolism and histidine turnover were elevated, including 3-hydroxyisovalerate to leucine (P < 0.05) and histamine to histidine (P < 0.05) (**Figure 1E**). Collectively, these findings confirm that the 20-week HFHS dietary intervention robustly induced hallmark features of obesity and diabetes, including obesity, glucose intolerance, insulin resistance, and significant disruptions in serum metabolite profiles.

### 3.2 Impact of Western diet on *A. aegypti* survival, reproduction, and lipid metabolism

**Figure 2:**
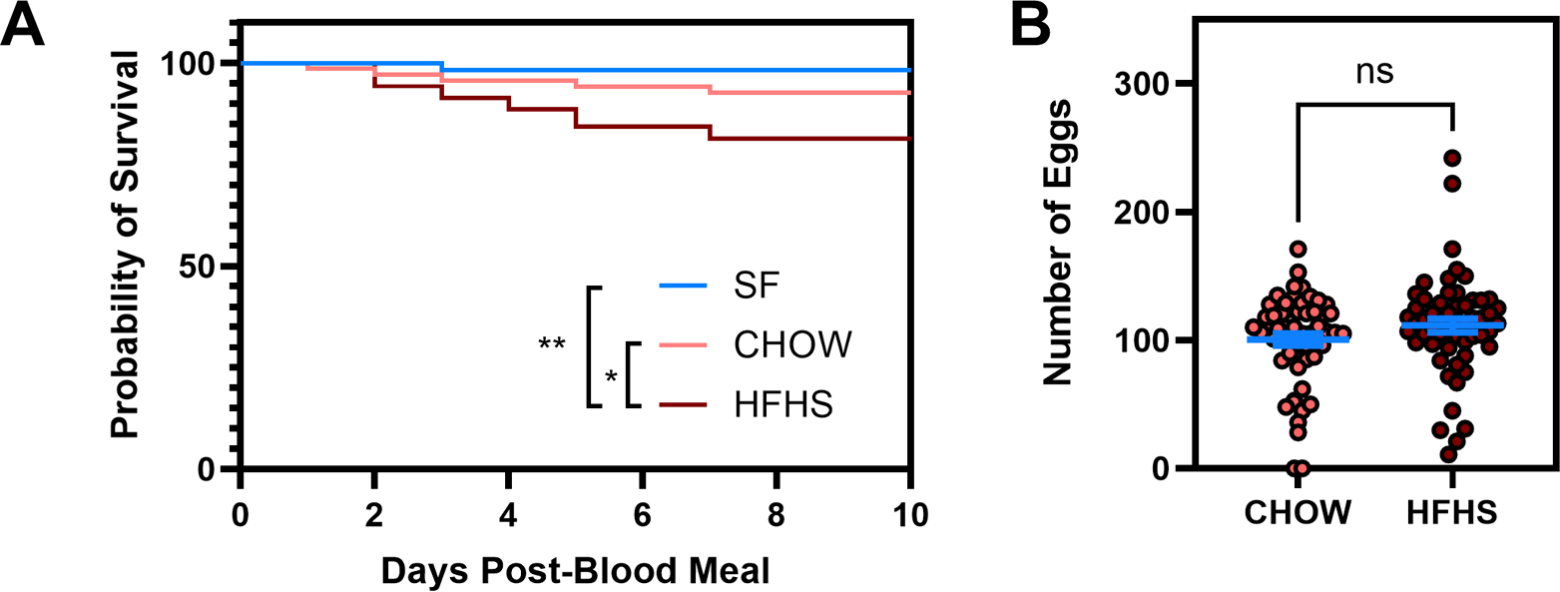
HFHS-derived blood meals increase early mosquito mortality without affecting total fecundity. *A. aegypti* mosquitoes were fed on AG129 mice subjected to either a CHOW or HFHS diet. Following blood feeding, either **(A)** mortality was tracked for 10 days, and the survival rate was compared against sugar-fed (SF) mosquitoes, or **(B)** mosquitoes were isolated in individual chambers and allowed to lay eggs. The number of eggs laid by individual mosquitoes was counted using a stereomicroscope. Statistical significance was determined using the Log-rank (Mantel-Cox) test for survival curve, and the Mann-Whitney unpaired test for oviposition was performed. Bars represent Mean +/- SEM, respectively. ns: not significant; *P<0.05; **P<0.01. A total of **(A)** SF: 55, CHOW: 69, HFHS: 70 mosquitoes, pooled from 2 independent mice feeding were used; and **(B)** CHOW: 58, HFHS: 56 mosquitoes pooled from 3 independent mice feeding were used.

To evaluate how host metabolic status affects mosquito fitness, we allowed 5-6-day-old female *A. aegypti* to feed directly on either anesthetized CHOW-fed mice (CHOW mosquitoes) or obese and diabetic mice (HFHS mosquitoes) after 20 weeks of dietary intervention. Both sugar-fed (SF) and CHOW-fed mosquitoes showed significantly better survival rates than HFHS-fed mosquitoes (Log-rank test, SF vs HFHS: P<0.01; CHOW vs HFHS: P<0.05) (**Figure 2A**). We further analyzed reproductive output, as a balance between survival and reproduction has been demonstrated in several models (47, 48). Mosquitoes were individually isolated in chambers and allowed to lay eggs at 5 dpbm. Despite the reduced survival, mosquitoes fed on CHOW mice showed no significant difference in the number of eggs laid compared to those fed on HFHS mice (CHOW: 100.4 ± 35.30 eggs, HFHS: 111.4 ± 40.03 eggs, Mann-Whitney test, P = 0.35) (**Figure 2B**).

**Figure 3:**
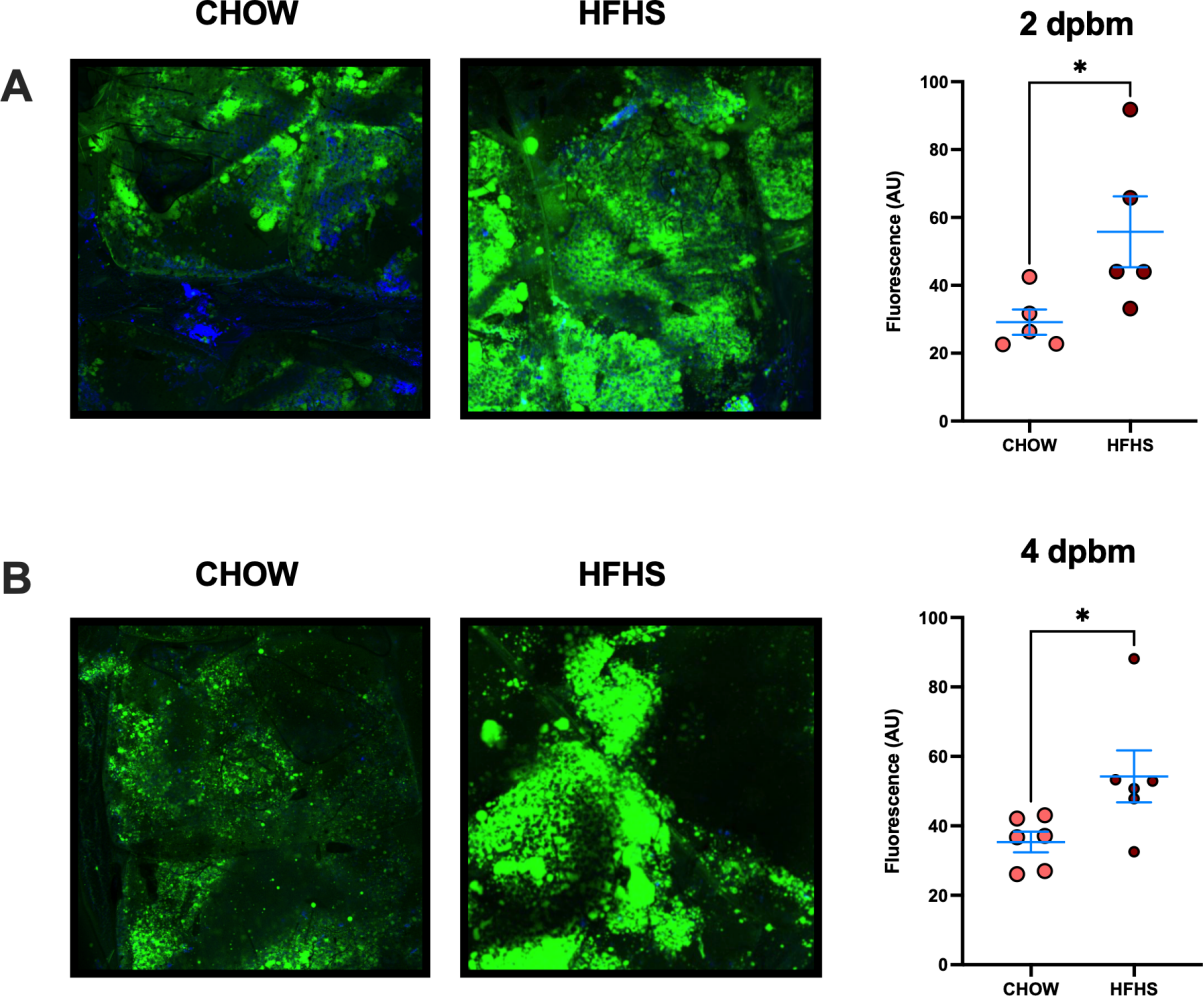
HFHS-derived blood meals lead to increased neutral lipid storage in the mosquito fat body. *Aedes aegypti* mosquitoes were fed on AG129 mice subjected to either a CHOW or HFHS diet. Following, fat body-enriched abdominal carcasses were dissected and incubated in 5 μM BODIPY 493/503 for labeling of neutral lipids. Imaging was performed using a confocal microscope and total BODIPY 493/503 fluorescence was posteriorly quantified, as described, at **(A)** 2 and **(B)** 4 dpbm. Each dot represents the normalized intensity of an individual mosquito fat body tissue. Statistical significance was determined using the unpaired t-test. Bars represent Mean +/- SEM, respectively *P < 0.05. A total of 3 independent mice feedings were performed. For panel **(A)**, 5 mosquitoes were analyzed per group (CHOW and HFHS). For panel **(B)**, 6 mosquitoes were analyzed per group.

The mosquito fat body functions as the primary organ for nutrient storage and metabolic regulation, making it a critical site for assessing lipid accumulation following blood feeding (49). To assess how host diet influences lipid storage in mosquitoes, we analyzed neutral lipid accumulation in the fat bodies of *A. aegypti* mosquitoes fed on either CHOW or HFHS mice. Fat body-enriched abdominal carcasses were dissected at 2 and 4 dpbm and stained with BODIPY 493/503 to visualize neutral lipids. At 2 dpbm, when lipid droplets are being replenished with blood meal-derived lipids (50), mosquitoes fed on CHOW mice showed significantly lower levels of neutral lipid storage compared to those fed on HFHS mice (CHOW: 29.17 ± 8.31 AU, HFHS: 55.77 ± 23.37 AU, P < 0.05) (**Figure 3A**). This trend continued at 4 dpbm, after complete termination of blood digestion (51), where CHOW-fed mosquitoes continued to exhibit less lipid accumulation than HFHS-fed mosquitoes (CHOW: 35.33 ± 7.29 AU, HFHS: 54.23 ± 18.30 AU, P < 0.05) (**Figure 3B**), corroborating that increased blood lipid levels results in higher lipid uptake and storage in mosquito fat bodies upon blood feeding.

**Figure 4:**
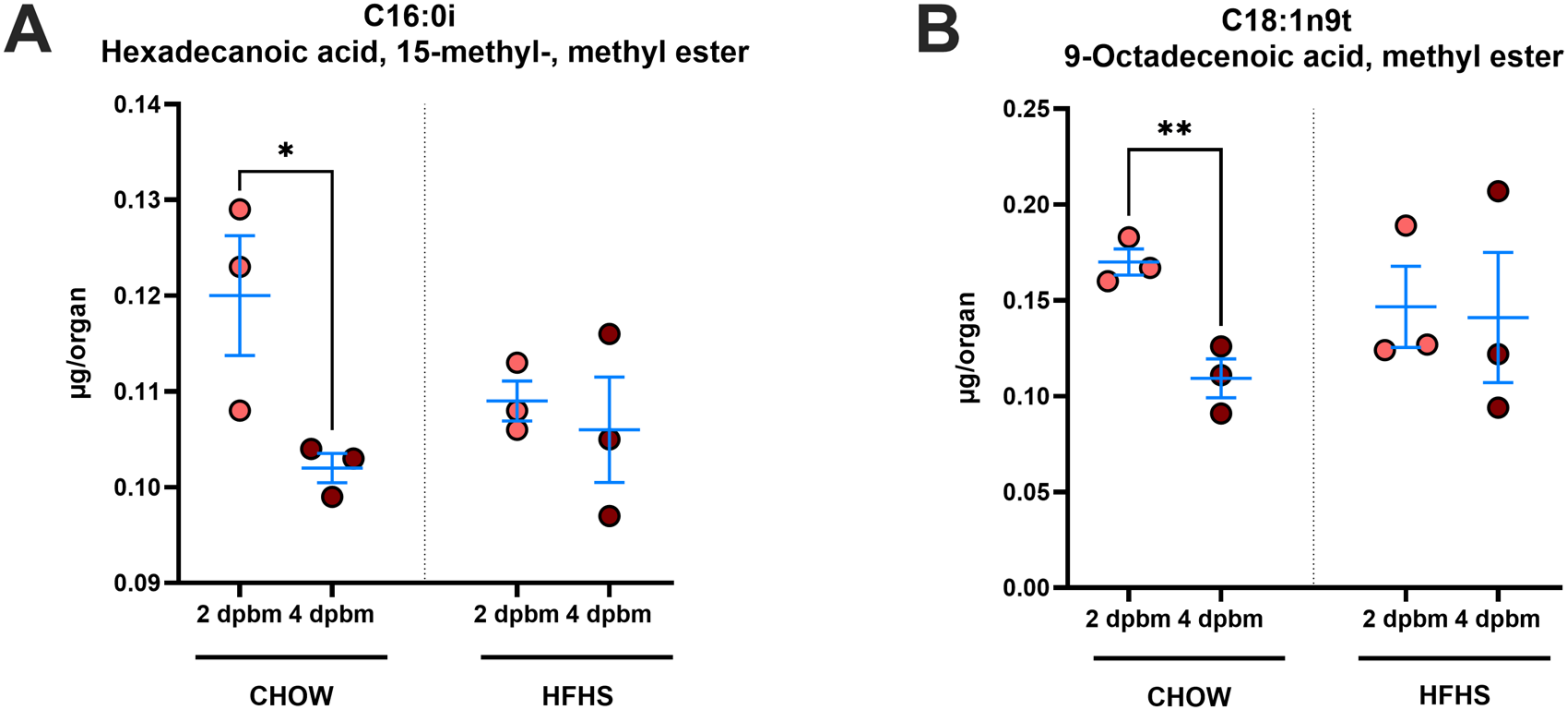
HFHS-derived blood meals mitigate post-feeding changes in specific fatty acid profiles. *Aedes aegypti* mosquitoes were fed on AG129 mice subjected to either a CHOW or HFHS diet. Following, fat body-enriched abdominal carcasses were dissected at 2 and 4 dpbm, and lipid content was extracted. Fatty acid content was analyzed by GC-MS, and the quantification of **(A)** Hexadecanoic acid, 15-methyl-, methyl ester (C16:0i), and **(B)** 9-Octadecenoic acid, methyl ester (C18:1n9t) is presented. Statistical significance was determined using the unpaired t-test. Bars represent Mean +/- SEM, respectively *P < 0.05, **P < 0.01. A total of 3 independent mice feeding were used for each condition (CHOW or HFHS). Each replicate was comprised of a batch of 15 pooled mosquitoes, which had been fed on an independent mouse.

To further investigate the impact of host diet on mosquito lipid metabolism, we analyzed the fatty acid composition of fat body-enriched abdominal carcasses at 1 and 4 dpbm using GC-MS. While PCA revealed no distinct separation between groups (**Supplementary Figure 1A**), the ingestion of blood from HFHS-fed mice mitigated some temporal changes in lipid composition observed after blood ingestion (**Supplementary Figure 1B**). Specifically, hexadecanoic acid, 15-methyl-, methyl ester (C16:0i) levels were significantly higher in CHOW mosquitoes at 2 dpbm compared to 4 dpbm (2dpbm: 0.1200 ± 0.011 µg/organ, 4 dpbm: 0.102 ± 0.002 µg/organ, P < 0.05), while no significant differences were observed in HFHS-fed mosquitoes (**Figure 4A**). Additionally, 9-octadecenoic acid, methyl ester (C18:1n9t) levels were significantly reduced at 4 dpbm in CHOW-fed mosquitoes compared to 2 dpbm (2dpbm: 0.170 ± 0.012 µg/organ, 4 dpbm: 0.109 ± 0.017 µg/organ, P < 0.01), whereas no significant changes occurred in HFHS-fed mosquitoes (**Figure 4B**).

### 3.3 Effects of vertebrate blood profile on midgut transcriptomic profiles

**Figure 5:**
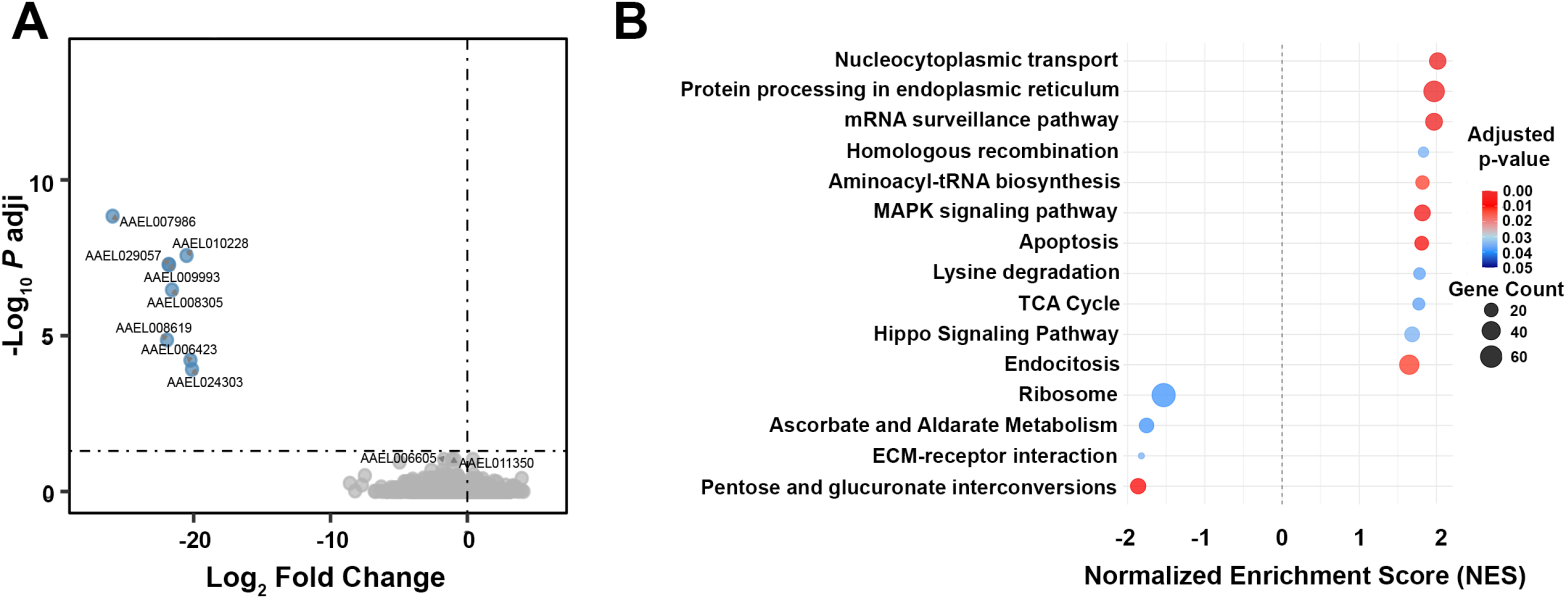
Naive HFHS blood meals induce subtle transcriptional changes but significantly alter metabolic and homeostatic pathways at 1 dpbm. *A. aegypti* mosquitoes were fed on AG129 mice subjected to either a CHOW or HFHS diet. Midgut samples were collected 1 dpbm, RNA was extracted, and samples were sent for transcriptome analysis. **(A)** Differential gene expression was assessed between CHOW-fed and HFHS-fed naive mosquitoes and represented in the volcano plot. Each dot represents a single gene. Genes significantly regulated in CHOW-fed mosquitoes are shown in light blue. Gray dots indicate non-significant genes. Dashed lines mark the significance thresholds for fold change and adjusted p-value (padj < 0.05, |log2FoldChange| > 0). **(B)** Enriched KEGG pathways were identified using GSEA. The Normalized Enrichment Score (NES) of differentially modulated pathways is plotted on the X-axis, with positive NES values representing upregulated pathways and negative NES values representing suppressed pathways in CHOW-fed naive mosquitoes. Bubble size represents the gene count (number of “core enrichment” genes that significantly contribute to the enrichment score), while color indicates adjusted p-value. A dashed vertical line at NES = 0 separates activated from suppressed pathways. Statistical significance for gene expression analysis was determined using DESeq2, and pathway enrichment was assessed using GSEA with KEGG database annotations. A total of 4 independent blood feedings were used per condition (CHOW or HFHS), where each replicate consisted of a pool of 15 mosquito midguts, which had been fed on an independent mouse.

To investigate the impact of host overweight and insulin resistance on *Aedes aegypti* physiology, we performed a transcriptomic analysis to compare non-infected (naive) mosquito midguts fed on either CHOW or HFHS mice (**Supplementary Table 3**). Our time points were chosen to capture two distinct and critical phases of this interaction. We analyzed 1 dpbm to capture the immediate transcriptional response during the peak of blood digestion (51) and intense metabolic activity, which is also the crucial window when the virus attempts to establish initial infections (52). In contrast, by 4 dpbm digestion is complete (51), but the infection is mostly restricted to the midgut (53). Interestingly, no gene was upregulated and only a small set of eight genes was found to be downregulated in naive CHOW-fed mosquitoes at 1 dpbm (**Figure 5A**, **Supplementary Table 4**). However, GSEA reveals significant regulation at the pathway-level, indicating that physiological adaptations are likely driven by subtle yet coordinated gene expression shifts across multiple functional pathways (**Figure 5B**). At 1 dpbm, genes involved in the citrate cycle (TCA cycle) and amino acid degradation were enriched in CHOW mosquitoes. Concomitantly, HFHS mosquitoes demonstrated increased pentose and glucuronate interconversions, and ascorbate and aldarate metabolism, potentially reflecting altered antioxidant activity. Apoptosis-related pathways were enriched in CHOW mosquitoes, suggesting increased cell death and cellular responses to blood digestion, which could lead to epithelial stress and potential barrier disruption. Accordingly, CHOW mosquitoes exhibited increased MAPK and Hippo signaling pathway activity, possibly as a compensatory mechanism for tissue turnover. In contrast, HFHS mosquitoes showed increased extracellular matrix (ECM)-receptor interaction pathways, suggesting structural remodeling of the midgut architecture.

At 4 dpbm, no significant differences in gene expression between CHOW and HFHS naive mosquitoes, suggesting an overall return of midgut metabolism to its pre-feeding state. However, some pathways remained altered (**Supplementary Figure 2**). Similar to the findings at 1 dpbm, HFHS mosquitoes exhibited increased ribosome activity and ribosome biogenesis, indicating sustained enhancement of protein translation activity. Interestingly, ABC transporters and immunity-related Toll and IMD signaling pathways were enriched in CHOW mosquitoes, possibly indicating a late-phase immune surveillance due to residual exposure to microbial or damage-associated molecular patterns (DAMPs) post-digestion.

### 3.4 Effects of vertebrate blood profile on *A. aegypti* midgut transcriptomic profiles during ZIKV infection

**Figure 6:**
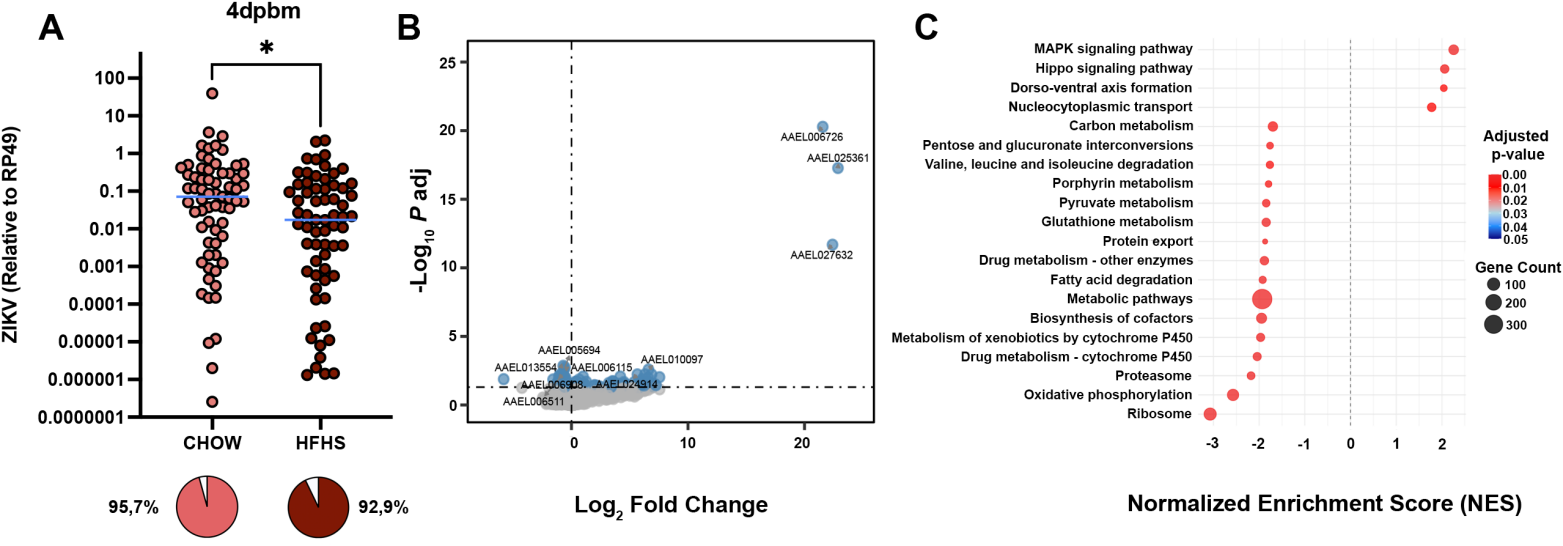
HFHS-derived blood meals reduce ZIKV infection intensity and correlate with broad metabolic and detoxification pathway enrichment at 1 dpbm. *A. aegypti* mosquitoes were fed with the blood of AG129 mice subjected to either CHOW or HFHS diets supplemented with ZIKV. **(A)** Midgut was dissected, and infection levels of infected mosquitoes were quantified by qRT-PCR 4 dpbm. Individual ZIKV RNA levels per infected mosquito (log scale) and total ZIKV prevalence were plotted as a pie chart. Furthermore, midgut samples were collected, RNA was extracted, and samples were sent for transcriptome analysis. **(B)** Differential gene expression was assessed between CHOW-fed and HFHS-fed infected mosquitoes 1 dpbm and represented in the volcano plot. Each dot represents a single gene. Genes significantly regulated in CHOW-fed mosquitoes are shown in light blue. Gray dots indicate non-significant genes. Dashed lines mark the significance thresholds for fold change and adjusted p-value (padj < 0.05, |log_2_FoldChange| > 0). **(C)** Enriched KEGG pathways were identified using GSEA. The Normalized Enrichment Score (NES) of differentially modulated pathways is plotted on the X-axis, with positive NES values representing upregulated pathways and negative NES values representing suppressed pathways in CHOW-fed infected mosquitoes. Bubble size represents the gene count (number of “core enrichment” genes that significantly contribute to the enrichment score), while color indicates adjusted p-value. A dashed vertical line at NES = 0 separates activated from suppressed pathways. ZIKV statistical analysis was performed using the Mann-Whitney unpaired test *: P<0.05; ZIKV prevalence was performed using the Chi-square test. Statistical significance for gene expression analysis was determined using DESeq2, and pathway enrichment was assessed using GSEA with KEGG database annotations. For panel **(A)**, a total of 70 individual mosquitoes per group were analyzed; these data were combined from 4 independent blood feedings. For panels **(B, C)**, 4 independent blood feedings were used per condition (CHOW or HFHS), where each replicate consisted of a pool of 15 mosquito midguts, which had been fed on an independent mouse.

To evaluate the effect of host diet on ZIKV infection dynamics in A. aegypti, mosquitoes were fed on the blood of AG129 mice subjected to either CHOW or HFHS diets supplemented with titrated ZIKV. At 4 dpbm, when infection remains mostly restricted to the gut (53), mosquitoes fed on CHOW mice exhibited significantly higher ZIKV infection intensity compared to those fed on HFHS mice (CHOW: Median 0.071 AU, HFHS: Median 0.017, Mann-Whitney test, P < 0.05) (**Figure 6A**). No difference in infection prevalence was found in these conditions (CHOW: 95,7%, HFHS: 92,9%). This reduction in infection intensity persisted in similar proportions at 8 dpbm (**Supplementary Figure 3A**), when virus has had sufficient time to disseminate from the midgut into the hemolymph (53, 54), with CHOW-fed mosquitoes showing significantly higher viral loads than HFHS-fed mosquitoes (CHOW: Median 0.72 AU, HFHS: Median 0.20 AU, Mann-Whitney test, P < 0.05). Restriction of virus replication at the midgut levels reflected in reduced systemic viremia, as corroborated by CHOW-fed mosquitoes showing higher ZIKV infection intensity in the abdominal carcass 8 dpbm compared to HFHS mosquitoes (CHOW: Median 0.01 AU, HFHS: Median 0.0002, Mann-Whitney test, P < 0.05) (**Supplementary Figure 3B**). Overall, these results demonstrate that blood from HFHS-fed mice reduces ZIKV infection and dissemination in A. aegypti.

To investigate whether early transcriptional responses contribute to this phenotype, we analyzed midguts at 1 dpbm, as early establishment of a refractory state might restrict viral replication and dissemination. Transcriptomic analysis of infected midguts at 1 dpbm revealed 249 differentially expressed genes between CHOW and HFHS infected mosquitoes (**Figure 6B**; **Supplementary Table 5**). To contextualize these transcriptomic differences, GSEA was performed to identify pathways differentially regulated in response to infection (**Figure 6C**). At 1 dpbm, infected HFHS mosquitoes exhibited significant enrichment of homeostatic, metabolic, and detoxification pathways. Oxidative phosphorylation was enriched in HFHS mosquitoes, suggesting increased mitochondrial activity. Similarly, pathways related to carbon metabolism, including amino acid degradation, fatty acid degradation, and pyruvate metabolism, were enriched in HFHS mosquitoes. Other metabolic pathways, including cytochrome P450 and glutathione metabolism, pentose and glucuronate interconversions, and porphyrin metabolism, were also upregulated in the HFHS group, highlighting an active detoxification process. In contrast, pathways associated with homeostatic regulation were diminished following HFHS feeding. The MAPK signaling and Hippo signaling pathways were significantly upregulated in CHOW mosquitoes, alongside Wnt signaling and dorso-ventral axis formation, possibly indicating active cellular turnover and tissue remodeling, as previously identified in naive mosquitoes.

At 4 dpbm, stress and immune-related pathways remained enriched in CHOW infected mosquitoes (**Supplementary Figure 4**). MAPK signaling, Hippo signaling, and dorso-ventral axis formation also remained enriched. Conversely, HFHS-fed infected mosquitoes continued to exhibit transcriptomic signatures centered on metabolism and detoxification, corroborating that a sustained early signature of blood-fed induced differences between CHOW and HFHS infected mosquitoes is maintained during the initial period of viral replication at the gut. Ribosome biogenesis, oxidative phosphorylation, and detoxification pathways (cytochrome P450 metabolism) remained enriched, as did glycine/serine/threonine metabolism and spliceosome-associated processes. This indicates persistent metabolic reprogramming for cellular maintenance and biosynthesis over immune activation.

## 4 Discussion

The midgut epithelium serves as the primary barrier to arbovirus dissemination (35, 36), yet this barrier is profoundly modulated by host-derived metabolic signals present in ingested blood. Prior studies have shown that single components, such as insulin or glucose, can modulate virus infection. However, the implications of a complex, diet-induced metabolic syndrome — which includes hyperinsulinemia, dyslipidemia, and altered amino acids — on vector capacity, metabolism and transcriptomic profile remain underexplored. Here, we addressed this gap using a holistic diet mimicking a Western Diet to assess these impacts on *A. aegypti* during ZIKV infections.

Following blood ingestion, host-derived molecules directly engage with gut cells and their receptors, initiating a cascade of physiological and immune responses. Insects have evolved sophisticated mechanisms to interpret these molecular signals, adapting their physiology to optimize survival and modulate infection dynamics. Notably, prior studies have demonstrated that insulin (24, 55) and glucose (21) exert opposing influences on arbovirus replication within mosquitoes; insulin enhances antiviral signaling pathways, whereas glucose facilitates viral replication. Similarly, cytokines, along with macro- and micronutrients, are known modulators of vector physiology, immunity, and metabolism upon blood feeding (22, 23, 25). Nevertheless, the implications of disrupted metabolic conditions in vertebrate hosts on vector physiology and viral replication remain underexplored. Here, we employed a 20-week HFHS dietary intervention designed to simulate the nutritional composition and gradual progression of metabolic alterations associated with Western Diets. This model recapitulates the human dietary patterns driving the global rise in obesity and comorbidities, such as insulin resistance and metabolic syndrome, offering greater physiological relevance than pharmacological and genetic models (56). The comprehensive physiological and metabolomic profiling of mice fed on an HFHS diet confirmed the development of insulin resistance, impaired glucose tolerance, hyperinsulinemia, and hyperglycemia, which, together with overweight, are associated with the development of metabolic syndrome. Additionally, choline levels were increased in HFHS-fed mice, suggesting dysregulation in lipid metabolism. Importantly, there was a marked reduction in circulating amino acid concentrations (tryptophan, leucine, and arginine) mirroring metabolomic patterns reported in other murine models of type II diabetes (57). Additionally, the histidine:histamine ratio was higher in HFHS-fed mice, indicating altered histidine metabolism, likely due to a chronic inflammatory state commonly caused by obesity (58).

Lipid acquisition post-blood ingestion is crucial for mosquito ovarian development and egg production (59). Accordingly, when mosquitoes were allowed to feed on hyperlipidemic HFHS mice (46), we observed increased fat body lipid content. This lipid storage phenotype was accompanied by subtle but consistent alterations in fatty acid mobilization post-blood feeding, as evidenced by changes in the relative abundance of C16:0i and C18:1n9t. While the impact of such differences in fatty acid mobilization remains to be explored, these findings gain relevance in the context of arboviral infections. Flaviviruses such as dengue virus extensively remodel host lipid metabolism to promote their replication (60). As such, eicosapentaenoic acid has recently been shown to have anti-ZIKV activity (61). Also, other unsaturated fatty acids have been shown to have antiviral activity (62). A transient metabolic shift was also reflected in the gut transcriptomic data, which identified downregulation of the TCA cycle 24h post-blood ingestion, suggesting a reduced reliance on oxidative energy metabolism by HFHS mosquitoes (63, 64). Despite increased lipid storage in the fat body, we did not observe a corresponding increase in the number of eggs laid per female. This is contrary to a previous observation of an increase in the total laid mass of eggs under similar conditions (28). Previous work has shown that blood-derived amino acids activate the Target-of-Rapamycin (TOR) signaling pathway and play a crucial role in vitellogenesis (65–67). Thus, the reduction of amino acid levels found in the blood of HFHS mice may counterbalance the increase in lipid storage and minimize effects on oogenesis. Further investigations are required to determine whether embryo viability and larval development are also affected.

Despite the transient nature of gut transcriptional responses, our data demonstrate that mosquitoes feeding on blood from HFHS-fed mice exhibit significantly reduced survival compared to those feeding on blood from CHOW-fed mice. While the anesthetic (ketamine/xylazine) used for feeding is a potential confounder, it has been shown to induce negligible effect on key life-history traits in *Anopheles* mosquitoes (68). This increased mortality might stem from enhanced oxidative stress, a known outcome of excessive insulin signaling in other models (69). While increased oxidative damage under these conditions remains to be experimentally confirmed, it is supported by heightened activity in the ascorbate and aldarate metabolic pathways. Further reinforcing this notion, we observed upregulation of the pentose and glucuronate interconversion pathways, potentially enhancing the mosquito’s capacity to counteract ROS via increased NADPH production through the pentose phosphate pathway—a well-established oxidative stress-response mechanism (70, 71). Detailed enrichment analysis of our dataset highlighted a putative role for several UDP-glucuronosyl/UDP-glucosyltransferases, NADP-dependent oxidoreductases, and alcohol dehydrogenases, suggesting the coordinated activation of detoxification, redox buffering, and sugar acid interconversion pathways. We also detected transcriptional regulation of ECM-receptor interaction pathways, likely in response to ROS and other cytotoxic byproducts generated during blood ingestion and digestion, as reported in several pathologies (72–74). Interestingly, ECM remodeling was negatively correlated with critical homeostatic signaling pathways involved in tissue integrity and regeneration, such as MAPK, Hippo, and apoptosis. This is noteworthy given accumulating evidence from mosquito and *Drosophila* models that highlights the importance of these pathways in gut regeneration under healthy physiological conditions (75, 76). Together, these findings suggest a scenario in which oxidative ECM damage occurs in the absence of compensatory regenerative signaling, potentially compromising tissue renewal and function. In the future, morphological studies should more closely examine ROS production and midgut architecture under these conditions to ascertain whether defective epithelial turnover contributes to tissue deterioration and increased mortality following blood feeding.

ZIKV infection intensity was consistently lower in the gut of HFHS mosquitoes at both 4 and 8 dpbm. This phenotype recapitulates the antiviral effects observed with insulin blood supplementation (24) and suggests that hyperinsulinemia signaling exerts a dominant influence on the vertebrate-invertebrate interface, potentially counteracting the proviral effects of other blood components such as glucose (21). This is further supported by the fact that insulin supplementation to the blood was found to increase mortality in *Anopheles*, similar to our findings upon HFHS bood feeding (77). Interestingly, insulin antiviral effects were suggested to be driven by ERK-induced JAK/SAT modulation (24). Accordingly, pathways such as MAPK and Hippo, which were found to be altered in our transcriptomic dataset, are known to interact with the JAK/STAT pathway in other insect models (78). This suggests that JAK/STAT could also be under regulation and playing a significant role in the observed reduction of infection levels in HFHS mosquitoes. More broadly, this highlights that alterations in blood composition associated with metabolic diseases may impair the mosquito’s ability to support viral replication and transmission.

Transcriptomic profiling suggests that ingestion of HFHS blood triggers a metabolic shift in the mosquito midgut that persists during infection and contributes to a refractory environment. When infected, HFHS mosquitoes exhibited sustained enrichment of pathways associated with energetic metabolism, particularly oxidative phosphorylation, consistent with recent evidence that mitochondrial activity and energy flux can limit viral replication (79, 80). This shift was accompanied by altered lipid and amino acid metabolism, further suggesting systemic reprogramming of midgut physiology. Additionally, HFHS-infected mosquitoes showed altered activation of pathways involved in cellular turnover and tissue regeneration, including MAPK, Hippo, apoptosis, Wnt, and dorso-ventral patterning. This may indicate compromised gut homeostasis and regeneration due to reduced viral load or damage signaling, similar to observations in naive states.

In parallel, we observed enrichment of pathways with putative antiviral and detoxification roles. Oxidative stress has been shown to exert protective effects against viral infection in mosquito models (80–82). Apart from pentose and glucuronate interconversion, increased activity of cytochrome P450 pathways may reflect elevated oxidative stress (83) or activation of detoxification (84) in response to tissue injury and infection. Notably, cytochrome P450 enzymes have documented antiviral roles (85), supporting the idea that their upregulation may enhance the mosquito’s antiviral defenses under HFHS conditions. Moreover, oxidative stress can induce protein degradation pathways, including proteasome activation (86). Antiviral functions of proteasomes have been proposed (87, 88), and recent studies further highlight their role in producing antimicrobial peptides in vertebrate models (89). Additional modulated pathways with putative antiviral relevance include butanoate metabolism, previously shown to amplify cytokine responses and antimicrobial peptide expression (90–92), and retinol metabolism, which is linked to epithelial integrity and oxidative stress mitigation (93). Interestingly, oxidative phosphorylation is essential for the activation of retinoic acid-inducible gene I-like receptors (94), suggesting a potential crosstalk between mitochondrial metabolism and retinol signaling in shaping mosquito antiviral responses.

In summary, this study demonstrates that diet-induced overweight and insulin resistance significantly alter mosquito physiology, reducing gut ZIKV infections intensity through mechanisms likely rooted in metabolic homeostasis and stress adaptation. While canonical antiviral pathways are well-established critical components in vector immunity, our data support a model in which metabolic reprogramming further enhances protection in HFHS mosquitoes. In this model, these alterations restrict viral replication by shaping the midgut environment through enhanced energetic metabolism, redox buffering, and translational regulation, rather than altered canonical immune activation. These metabolic shifts appear to create a midgut environment that is not only less amenable to infection but may also impact homeostatic regeneration, as evidenced by the regulation of signaling pathways essential for epithelial renewal and increased mortality. The interplay between mitochondrial activity, oxidative stress, and tissue integrity thus emerges as a critical determinant of vector immunity under conditions of host-derived metabolic stress. Future studies should use functional assays and reverse genomics to further elucidate the interplay between these pathways in shaping the mosquito’s antiviral response.

The present study aimed to evaluate the effects of altered host metabolism on the transcriptional response and susceptibility of the gut to arboviral infections. Future research endeavors should investigate the impact of analogous dietary models on viral transmission dynamics, including infections within the salivary glands and transmission to murine models. These findings may underscore the necessity of incorporating host nutritional status into models of vector-borne disease transmission, including its impact on infection life expectancy, susceptibility, fecundity, and larval development. This issue becomes more complex as host viremia is also shaped by its metabolic status during arboviral infections (95, 96). It is also important to emphasize that our findings—namely, that blood from a metabolically dysregulated host reduces mosquito viral susceptibility—should not be misinterpreted as suggesting that obesity is protective at the host or population level.

That host diet can modulate mosquito susceptibility to infection raises the possibility that rising rates of obesity and metabolic syndrome, particularly in low- and middle-income countries (97), may influence arbovirus transmission dynamics. As of 2022, over 1 billion people worldwide are estimated to be living with obesity, including over 850 million adults, with prevalence accelerating most rapidly in LMICs (33). This trend has reached alarming levels in many regions across Latin America, Asia, and Africa (98), overlapping geographically with areas of high arboviral burden, raising concern that changes in host nutritional status could alter the landscape of vector-borne disease transmission. Understanding how metabolic diseases in reservoir hosts shape vector physiology will be critical for improving disease risk prediction.

## Data Availability

The datasets presented in this study can be found in online repositories. The names of the repository/repositories and accession number(s) can be found in the article/**Supplementary Material**.
