# Supplementary material for: A diet-induced obese and diabetic host phenotype reduces mosquito ZIKV infections and remodels gut metabolism
Source: Front Immunol. 2025 Dec 15;16:1704301. doi: 10.3389/fimmu.2025.1704301 (PMC12745224; doi:10.3389/fimmu.2025.1704301)
Supplement: Supplementary file 1 [file DataSheet1.pdf]

A Diet-Induced Obese and Diabetic Host Phenotype Reduces Mosquito ZIKV Infections and Remodels Gut Metabolism

Supplemental Information

CHOW DIET	RATIO
Corn Starch	46.200%
Casein	16.500%
Dextrinized Starch	13.000%
Sucrose	10.000%
Soybean Oil	4.000%
Microcrystalline Cellulose	5.000%
Mineral Mix specific for AIN 93M diets	3.500%
Vitamin Mix specific for AIN 93M diets	1.300%
L-Cystine	0.250%
Choline Bitartrate	0.250%

HFHS DIET	RATIO
Casein	20.950%
Sucrose	27.050%
Soybean Oil	4.000%
Microcrystalline Cellulose	5.000%
AIN 93G Mineral Mix	3.500%
AIN 93 Vitamin Mix	1.300%
L-Cystine	0.300%
Choline Bitartrate	0.250%
Lard	37.650%

Supplemental Table 1. Diet Composition. Mice were fed either a high-fat high-sucrose (HFHS) diet or a standard chow (CHOW) diet for 20 weeks to induce metabolic syndrome symptoms. Composition of diet is specified.

Parameter	CHOW	HFHS	P-value
**Total body weight gain (g)**	12.53±2.82	18.76±6.04	P<0.001
**Insulin tolerance test (auc, au)**	10.169±4.264	13.349±2.763	P<0.05
**Oral glucose tolerance test (auc, au)**	13.313±5.520	18.139±6.585	P<0.001
**Resting serum insulin (µui/ml)**	13.01±1.00	25.34±1.07	P<0.0001

Supplemental Table 2: Summary of key metabolic parameters from AG129 mice after 20-week dietary intervention. Mice were either fed a high-fat, high-sucrose (HFHS) diet or a standard chow (CHOW) diet for 20 weeks**.** Data are presented as Mean ± SEM. Sample sizes were 18 mice per group for Total Weight Gain, ITT, and oGTT, and 8 mice per group for Resting Insulin. Statistical significance was determined using an unpaired t-test.

Supplemental Table 3. Quality Control Metrics and Alignment Statistics for RNA-Seq Libraries. Total RNA was extracted from individual midgut pools (n=4 per condition), and library integrity was verified (RIN > 7) prior to sequencing on the Illumina NextSeq 2000 platform. Raw sequencing reads were processed for quality control, trimming, and alignment to the *Aedes aegypti* reference genome (AaegL5) using the STAR aligner. The table presents key metrics for each sample, including: (1) Sample Metadata (ID, diet, infection status, and time point); (2) Sequencing Quality (RNA Integrity Number and raw read counts); (3) Data Processing (number and percentage of reads after trimming); and (4) Alignment & Quantification (number and percentage of uniquely mapped reads and reads successfully assigned to gene features).

Supplemental Table 3. Quality Control Metrics and Alignment Statistics for RNASeq Libraries

							Trimming			Mapping		Counting			
Sample	ID	dpbm	Diet	ZIKA infected	RIN	number of raw reads	number of trimmed reads	percent of trimmed reads	Q30 (%)	uniquely mapped reads	percent	no feature	ambiguous	feature	percent
I1CI1	52- Exp3 Mg 1d C	1	CHOW	Yes	10.0	16.347.592	15.384.260	94,1%	94,2	12.995.713	84,5%	668.103	65.411	12.262.199	94,4%
I1CI2	67 - Exp4 Mg 1d C	1	CHOW	Yes	10.0	16.371.311	15.277.967	93,3%	93,93	13.186.646	86,3%	627.563	62.768	12.496.315	94,8%
I1CI3	82 - Exp5 Mg 1d C	1	CHOW	Yes	10.0	16.192.267	15.250.699	94,2%	93,86	12.873.091	84,4%	585.158	66.111	12.221.822	94,9%
I1CI4	92- Exp6 Mg 1d C	1	CHOW	Yes	10.0	15.754.238	14.747.385	93,6%	93,98	12.360.294	83,8%	429.465	57.830	11.872.999	96,1%
I1CN1	51 - Exp3 Mg 1d B	1	CHOW	No	9.8	17.122.971	15.290.246	89,3%	94,03	12.944.823	84,7%	645.984	70.298	12.228.541	94,5%
I1CN2	66 - Exp4 Mg 1d B	1	CHOW	No	9.9	15.327.914	14.324.552	93,5%	94,13	12.418.010	86,7%	632.611	62.345	11.723.054	94,4%
I1CN3	81 - Exp5 Mg 1d B	1	CHOW	No	9.9	19.596.866	17.037.447	86,9%	93,92	14.145.286	83,0%	537.933	78.604	13.528.749	95,6%
I1CN4	91- Exp6 Mg 1d B	1	CHOW	No	10.0	16.904.667	16.145.686	95,5%	94,13	13.802.749	85,5%	580.088	69.745	13.152.916	95,3%
I1HI1	54 - Exp3 Mg 1d E	1	HFHS	Yes	10.0	15.737.687	15.095.280	95,9%	94,04	12.269.489	81,3%	353.523	69.278	11.846.688	96,6%
I1HI2	69 - Exp4 Mg 1d E	1	HFHS	Yes	10.0	17.379.650	15.649.906	90,0%	93,92	13.241.198	84,6%	363.277	68.299	12.809.622	96,7%
I1HI3	84 - Exp5 Mg 1d E	1	HFHS	Yes	9.6	19.296.919	12.431.306	64,4%	93,84	10.337.715	83,2%	457.527	54.840	9.825.348	95,0%
I1HI4	94 - Exp6 Mg 1d E	1	HFHS	Yes	10.0	16.487.060	15.252.427	92,5%	93,88	12.634.207	82,8%	544.913	65.204	12.024.090	95,2%
I1HN1	53 - Exp3 Mg 1d D	1	HFHS	No	9.1	12.974.985	11.936.576	92,0%	93,91	10.089.912	84,5%	559.712	62.617	9.467.583	93,8%
I1HN2	68 - Exp4 Mg 1d D	1	HFHS	No	9.7	17.274.863	15.220.746	88,1%	94,12	13.151.446	86,4%	567.899	72.315	12.511.232	95,1%
I1HN3	83 - Exp5 Mg 1d D	1	HFHS	No	10.0	15.326.440	14.739.031	96,2%	94,14	12.053.775	81,8%	598.977	68.960	11.385.838	94,5%
I1HN4	93- Exp6 Mg 1d D	1	HFHS	No	10.0	16.911.230	16.101.758	95,2%	94,13	13.188.762	81,9%	434.262	65.757	12.688.743	96,2%
I4CI1	57 - Exp3 Mg 4d C	4	CHOW	Yes	10.0	15.765.390	15.209.276	96,5%	93,97	13.583.068	89,3%	739.793	89.360	12.753.915	93,9%
I4CI1	97 - Exp6 Mg 4d C	4	CHOW	Yes	10.0	15.139.397	14.486.624	95,7%	94,13	12.905.426	89,1%	663.919	87.824	12.153.683	94,2%
I4CI2	72 - Exp4 Mg 4d C	4	CHOW	Yes	10.0	14.742.192	13.854.081	94,0%	93,97	12.281.379	88,6%	697.284	82.308	11.501.787	93,7%
I4CI3	87 - Exp5 Mg 4d C	4	CHOW	Yes	9.8	15.453.113	14.926.981	96,6%	93,95	13.141.325	88,0%	805.008	94.332	12.241.985	93,2%
I4CN1	56 - Exp3 Mg 4d B	4	CHOW	No	9.9	15.705.424	15.175.625	96,6%	94,01	13.530.886	89,2%	714.829	89.955	12.726.102	94,1%
I4CN2	71 - Exp4 Mg 4d B	4	CHOW	No	10.0	17.584.662	16.918.352	96,2%	94,12	15.145.377	89,5%	825.822	102.658	14.216.897	93,9%
I4CN3	86 - Exp5 Mg 4d B	4	CHOW	No	10.0	14.466.771	13.910.139	96,2%	94,19	12.129.734	87,2%	794.247	91.533	11.243.954	92,7%
I4CN4	96 - Exp6 Mg 4d B	4	CHOW	No	10.0	12.269.538	11.842.029	96,5%	93,76	10.527.713	88,9%	572.555	72.198	9.882.960	93,9%
I4HI2	74 - Exp4 Mg 4d E	4	HFHS	Yes	10.0	12.665.595	11.963.721	94,5%	94,18	10.335.224	86,4%	670.698	72.610	9.591.916	92,8%
I4HI3	89 - Exp5 Mg 4d E	4	HFHS	Yes	10.0	17.454.917	16.660.725	95,5%	93,5	14.790.633	88,8%	825.135	114.359	13.851.139	93,6%
I4HI4	59 - Exp3 Mg 4d E	4	HFHS	Yes	9.7	18.211.458	17.571.129	96,5%	94,15	15.634.601	89,0%	830.031	119.821	14.684.749	93,9%
I4HI4	99 - Exp6 Mg 4d E	4	HFHS	Yes	10.0	14.486.991	13.475.874	93,0%	94,23	11.828.878	87,8%	629.183	83.141	11.116.554	94,0%
I4HN1	58 - Exp3 Mg 4d D	4	HFHS	No	10.0	16.121.679	15.608.726	96,8%	93,99	13.896.832	89,0%	699.848	90.556	13.106.428	94,3%
I4HN2	73 - Exp4 Mg 4d D	4	HFHS	No	10.0	18.992.563	17.785.168	93,6%	94,01	15.448.951	86,9%	1.039.440	111.692	14.297.819	92,5%
I4HN3	88 - Exp5 Mg 4d D	4	HFHS	No	10.0	16.520.671	15.888.668	96,2%	94,08	13.899.721	87,5%	786.062	100.954	13.012.705	93,6%
I4HN4	98 - Exp6 Mg 4d D	4	HFHS	No	10.0	15.714.225	15.188.841	96,7%	93,77	13.258.147	87,3%	719.208	97.748	12.441.191	93,8%

Supplemental Table 4. Differentially Expressed Genes in the Midgut of Naive *A. aegypti* Mosquitoes. Mosquitoes were fed on AG129 mice subjected to either a CHOW or HFHS diet, and midguts were collected 1 day post-blood meal (dpbm) for transcriptomic analysis. Differentially expressed genes were identified using DESeq2, with significance determined based on adjusted p-value ($padj < 0.05$) and $|log_2FoldChange| > 1$ of CHOW-fed compared against HFHS-fed mosquitoes. Gene ontology (GO) annotations and protein domain classifications are provided. A total of 4 independent blood feedings were used per condition (CHOW or HFHS), where each replicate consisted of a pool of 15 mosquito midguts.

Supplemental Table 4. Differentially Expressed Genes in the Midgut of Naive A. aegypti Mosquitoes

GeneID	baseMean	log2FoldChange	lfcSE	stat	pvalue	padj	Product Description	1&%, 3URGXFW 'HVFULSWLRQ	Computed GO Processes	Computed GO Functions	3)$0
AAEL007986	137.99	-25.92	3.49	-7.42	1.18E-13	1.47E-09	unspecified product	1$	N/A	N/A	1 $
AAEL008619	19.79	-21.95	3.78	-5.80	6.61E-09	1.37E-05	unspecified product		proteolysis	serine-type endopeptidase activity	6HULQH SURWHDVHV WU\SVLQ GRPDLQ
AAEL029057	18.98	-21.83	3.22	-6.77	1.24E-11	5.17E-08	unspecified product		N/A	N/A	7R[6*6 GRPDLQ
AAEL009993	18.10	-21.80	3.24	-6.73	1.69E-11	5.27E-08	Tox-SGS domain-containing protein [Source:UniProtKB/TrEMBL;Acc:A0A1S4FPC9]		N/A	N/A	7R[6*6 GRPDLQ
AAEL008305	11.08	-21.59	3.36	-6.42	1.36E-10	3.39E-07	Putative 7.8 kDa acidic protein [Source:UniProtKB/TrEMBL;Acc:Q8T9V8]	1$	N/A	N/A	1 $
AAEL010228	26.58	-20.52	2.96	-6.93	4.28E-12	2.67E-08	unspecified product	N'D VDOLYDU\ JODQG DOOHUJHQ $HG D OLNH	N/A	N/A	1 $
AAEL006423	17.18	-20.24	3.67	-5.52	3.44E-08	6.12E-05	Short form D7Cclu23 salivary protein [Source:UniProtKB/TrEMBL;Acc:Q95V89]	1$	N/A	odorant binding	1 $
AAEL024303	8.43	-20.12	3.74	-5.37	7.72E-08	1.20E-04	unspecified product	1$	N/A	odorant binding	1 $

Supplemental Table 5. Differentially Expressed Genes in the Midgut of ZIKV-Infected *A. aegypti* Mosquitoes. Mosquitoes were fed on AG129 mice subjected to either a CHOW or HFHS diet, and midguts were collected 1 day post bloodmeal (dpbm) for transcriptomic analysis. Differentially expressed genes were identified using DESeq2, with significance determined based on adjusted p-value ($padj < 0.05$) and $|log_2FoldChange| > 1$ of CHOW-fed compared against HFHS-fed mosquitoes. Gene ontology (GO) annotations and protein domain classifications are provided. A total of 4 independent blood feedings were used per condition (CHOW or HFHS), where each replicate consisted of a pool of 15 mosquito midguts.

Supplemental Table 5. Differentially Expressed Genes in the Midgut of ZIKV-Infected A. aegypti Mosquitoes

GeneID	baseMean	g2FoldChang	lfcSE	stat	pvalue	padj	Product Description	NCBI Description	Computed GO Functions	Computed GO Processes	PFAM
AAEL025361	75.13	22.87	2.39	9.56	1.14E-21	5.32E-18	unspecified product	NA	N/A	N/A	N/A
AAEL027632	53.89	22.40	2.77	8.08	6.62E-16	2.06E-12	unspecified product	proteoglycan 4-like	N/A	N/A	N/A
AAEL006726	18.57	21.56	2.09	10.32	5.62E-25	5.25E-21	Innexin [Source:UniProtKB/TrEMBL;Acc:Q174Z8]		N/A	ion transport	Innexin
AAEL002422	48.60	7.55	1.84	4.10	4.13E-05	9.41E-03	cytoplasmic polyadenylation element binding protein (cpeb)		mRNA 3'-UTR binding;nucleic acid	regulation of translation	Cytoplasmic polyadenylation element-binding protein, ZZ domain;RNA
AAEL018263	36.17	7.25	2.14	3.39	7.01E-04	3.62E-02	unspecified product		N/A	N/A	OTU domain
AAEL019583	101.46	7.15	2.05	3.49	4.82E-04	3.03E-02	unspecified product		RNA binding;nucleic acid binding;	N/A	K Homology domain, type 1;Sterile alpha motif domain
AAEL000739	27.19	6.97	2.15	3.24	1.18E-03	4.68E-02	unspecified product		protein binding	N/A	F-box domain
AAEL004390	126.05	6.87	1.54	4.46	8.28E-06	5.87E-03	heme peroxidase		heme binding;peroxidase activity	cellular oxidant detoxification;obsolete	Haem peroxidase, animal-type
AAEL019551	30.53	6.72	1.88	3.58	3.46E-04	2.59E-02	unspecified product	NA	N/A	N/A	N/A
AAEL002652	117.62	6.63	1.79	3.71	2.08E-04	2.09E-02	unspecified product		N/A	N/A	Eukaryotic translation initiation factor 4E binding protein
AAEL010097	544.92	6.61	1.39	4.76	1.95E-06	2.59E-03	unspecified product		RNA binding;protein homodimerization	bicoid mRNA localization	Exuperantia, SAM-like domain
AAEL007657	109.74	6.59	1.63	4.03	5.54E-05	1.06E-02	low-density lipoprotein receptor (ldl)		calcium ion binding;protein binding	N/A	Low-density lipoprotein (LDL) receptor class A repeat;LDLR class B repeat;
AAEL010229	14.24	6.47	1.79	3.62	2.98E-04	2.38E-02	unspecified product	NA	N/A	N/A	N/A
AAEL019574	11.23	6.38	1.78	3.59	3.30E-04	2.54E-02	unspecified product		ATP binding	N/A	SNF2-related, N-terminal domain;Helicase, C-terminal
AAEL002036	32.16	6.36	1.93	3.29	9.88E-04	4.35E-02	unspecified product		metal ion binding	N/A	Tudor domain;Zinc finger, MYND-type
AAEL008829	49.79	6.36	1.76	3.62	2.91E-04	2.38E-02	unspecified product	fs(1)N female sterile (1) Nasrat	N/A	N/A	N/A
AAEL017403	286.68	6.30	1.41	4.48	7.54E-06	5.87E-03	Trypsin Modulating Oostatic Factor	vitelline membrane protein 1Sa-2	hormone activity	negative regulation of endopeptidase activity;	N/A
AAEL004386	127.93	6.25	1.85	3.38	7.34E-04	3.70E-02	chorion peroxidase		heme binding;metal ion binding;	cellular oxidant detoxification;hydrogen	Haem peroxidase, animal-type
AAEL027438	6.26	6.17	1.88	3.28	1.03E-03	4.41E-02	unspecified product	NA	N/A	N/A	N/A
AAEL000442	234.27	6.08	1.53	3.96	7.45E-05	1.29E-02	protein oskar		N/A	N/A	OSK domain
AAEL003404	141.71	6.04	1.60	3.78	1.59E-04	1.88E-02	unspecified product	NA	N/A	N/A	N/A
AAEL012708	11.50	6.00	1.75	3.44	5.87E-04	3.34E-02	unspecified product		RNA binding;nucleic acid binding	N/A	K Homology domain, type 1
AAEL001666	119.32	5.99	1.81	3.30	9.61E-04	4.27E-02	unspecified product		N/A	regulation of gene expression	OST-HTH/LOTUS domain
AAEL011470	37.85	5.91	1.68	3.52	4.34E-04	2.81E-02	cis,cis-muconate transport protein MucK, putative		transmembrane transporter activity	transmembrane transport	MaMor facilitator superfamily
AAEL026038	43.81	5.82	1.81	3.21	1.32E-03	4.98E-02	unspecified product		heme binding;peroxidase activity	cellular oxidant detoxification;obsolete	Haem peroxidase, animal-type
AAEL017098	727.52	5.78	1.63	3.54	4.05E-04	2.78E-02	unspecified product		iron ion binding;metal ion binding;	aromatic amino acid family metabolic process;	Aromatic amino acid hydroxylase, C-terminal
AAEL000923	56.70	5.75	1.57	3.66	2.56E-04	2.23E-02	unspecified product		N/A	N/A	EB domain
AAEL000776	76.38	5.72	1.56	3.67	2.47E-04	2.22E-02	unspecified product		zinc ion binding	N/A	B-box-type zinc finger
AAEL024914	126.99	5.65	1.27	4.47	8.00E-06	5.87E-03	Putative synaptic vesicle transporter svop [Source:		transmembrane transporter activity	transmembrane transport	MaMor facilitator, sugar transporter-like
AAEL012015	14.13	5.57	1.61	3.46	5.36E-04	3.19E-02	DEAD box ATP-dependent RNA helicase		ATP binding;nucleic acid binding	N/A	DEAD/DEAH box helicase domain;Helicase, C-terminal
AAEL013338	342.39	5.37	1.46	3.67	2.41E-04	2.19E-02	lethal(2)essential for life protein, l2efl		metal ion binding	N/A	Alpha crystallin/Hsp20 domain
AAEL007097	707.90	4.80	1.43	3.36	7.89E-04	3.78E-02	4-nitrophenylphosphatase [Source:UniProtKB/TrEMBL;Acc:		hydrolase activity	N/A	HAD-superfamily hydrolase, subfamily IIA
AAEL014365	37.54	4.63	1.42	3.27	1.09E-03	4.49E-02	chromaffin granule amine transporter, putative		transmembrane transporter activity	transmembrane transport	MaMor facilitator superfamily
AAEL019770	13.42	4.55	1.30	3.51	4.44E-04	2.82E-02	unspecified product		DNA binding;DNA-binding transcription	regulation of transcription, DNA-templated	Homeobox domain;POU-specific domain
AAEL002228	31.71	4.50	1.22	3.68	2.32E-04	2.13E-02	fatty acid synthase		catalytic activity;fatty acid synthase	biosynthetic process;obsolete oxidation-	Beta-ketoacyl synthase, N-terminal;Phosphopantetheine binding ACP
AAEL000961	47.19	4.36	1.33	3.27	1.06E-03	4.49E-02	unspecified product	NA	metal ion binding	N/A	N/A
AAEL016992	9.66	4.19	0.99	4.22	2.49E-05	8.40E-03	unspecified product		copper ion binding;metal ion binding;	obsolete oxidation-reduction process	Multicopper oxidase, type 1;Multicopper oxidase, type 2;Multicopper
AAEL006890	78.51	4.02	1.14	3.52	4.27E-04	2.81E-02	unspecified product		N/A	N/A	Protein of unknown function DUF4820
AAEL008046	19.28	3.62	1.03	3.52	4.28E-04	2.81E-02	rh antigen		ammonium transmembrane	ammonium transmembrane transport;	Ammonium transporter AmtB-like domain
AAEL011532	30.10	3.60	1.09	3.30	9.51E-04	4.25E-02	unspecified product	NA	N/A	N/A	N/A
AAEL002554	67.10	3.58	0.96	3.73	1.94E-04	2.00E-02	anosmin, putative		peptidase inhibitor activity;protein	N/A	WAP-type 'four-disulfide core' domain
AAEL009411	20.04	3.52	0.92	3.81	1.41E-04	1.82E-02	DNA-binding protein smubp-2		S'-3' RNA helicase activity;RNA	post-transcriptional gene silencing by RNA	DNA2/NAM7 helicase-like, AAA domain
AAEL005131	10.14	3.49	1.01	3.45	5.69E-04	3.26E-02	protein kinase c-binding protein nell1		calcium ion binding;protein binding	N/A	9WFC domain;EGF-like calcium-binding domain;EGF domain
AAEL027536	30.24	3.40	1.05	3.23	1.25E-03	4.86E-02	Putative alpha amylase catalytic domain family found in		N/A	N/A	Solute carrier family 3 member 2, N-terminal domain
AAEL008559	26.87	3.20	0.89	3.60	3.22E-04	2.51E-02	glutaminase		glutaminase activity;protein binding	glutamine metabolic process	Glutaminase;Ankyrin repeat-containing domain;Glutaminase, EF-hand
AAEL022657	11.22	2.44	0.75	3.27	1.07E-03	4.49E-02	unspecified product		acyltransferase activity, transferring	N/A	Acyltransferase 3
AAEL014053	65.43	2.18	0.65	3.33	8.62E-04	4.00E-02	vacuolar proton ATPases		proton transmembrane transporter	ion transport;proton transmembrane transport	9-type ATPase, 90 complex, 116kDa subunit family
AAEL023413	91.34	1.99	0.60	3.34	8.25E-04	3.89E-02	unspecified product		nucleic acid binding	N/A	Zinc finger C2H2-type
AAEL005281	40.87	1.98	0.58	3.40	6.86E-04	3.58E-02	unspecified product		N/A	N/A	Ima1, N-terminal domain
AAEL010469	62.18	1.26	0.34	3.73	1.88E-04	2.00E-02	MACPF domain-containing protein [Source:		N/A	N/A	Membrane attack complex component/perforin (MACPF) domain
AAEL019863	92.49	1.15	0.33	3.47	5.24E-04	3.19E-02	unspecified product		DNA binding;DNA-binding transcription	regulation of transcription, DNA-templated	Nuclear hormone receptor, ligand-binding domain;Zinc finger, nuclear
AAEL011413	49.54	1.07	0.30	3.54	4.01E-04	2.77E-02	unspecified product		protein binding;ubiquitin protein ligase	protein ubiquitination	RWD domain
AAEL011993	42.21	1.01	0.30	3.36	7.87E-04	3.78E-02	unspecified product		DNA binding;nuclease activity	nucleic acid phosphodiester bond hydrolysis	ERCC4 domain
AAEL000116	37.30	1.00	0.24	4.19	2.85E-05	8.40E-03	Anaphase-promoting complex subunit 4 [Source:		protein binding	anaphase-promoting complex-dependent	Anaphase-promoting complex subunit 4, WD40 domain;Anaphase-
AAEL004243	62.26	0.99	0.29	3.36	7.86E-04	3.78E-02	S1 RNA binding domain protein, putative		nucleic acid binding	nucleobase-containing compound metabolic	S1 domain;Tex-like protein, N-terminal;Tex protein, YqgF-like domain;HHH
AAEL008773	63.75	0.97	0.29	3.36	7.78E-04	3.78E-02	laminin A chain, putative		N/A	cell adhesion	Laminin I9;Laminin EGF domain;Laminin, N-terminal;Laminin G domain;
AAEL025042	55.23	0.85	0.26	3.32	9.15E-04	4.18E-02	unspecified product		3'-S' DNA helicase activity;ATP	DNA duplex unwinding;DNA recombination;	DEAD/DEAH box helicase domain;Helicase, C-terminal;HRDC domain;
AAEL011201	149.55	0.72	0.22	3.29	1.01E-03	4.40E-02	unspecified product		N/A	N/A	Domain of unknown function DUF2428, death-receptor-like
AAEL008557	192.07	0.65	0.17	3.92	8.94E-05	1.39E-02	unspecified product		structural constituent of nuclear pore	nucleocytoplasmic transport	Nucleoporin Nup188
AAEL000703	872.16	0.65	0.19	3.45	5.55E-04	3.24E-02	glycogen phosphorylase		1,4-alpha-oligoglucan phosphorylase	carbohydrate metabolic process	Glycosyl transferase, family 3S
AAEL003032	227.21	0.64	0.20	3.28	1.04E-03	4.43E-02	PHD finger protein		metal ion binding	N/A	Zinc finger, PHD-finger
AAEL004117	414.89	0.52	0.16	3.35	8.04E-04	3.83E-02	ATP-dependent RNA helicase		ATP binding;helicase activity;	N/A	DEAD/DEAH box helicase domain;Helicase, C-terminal;R3H domain;
AAEL007252	494.77	0.47	0.13	3.68	2.32E-04	2.13E-02	sin3a-associated protein sap130		N/A	regulation of transcription, DNA-templated	Histone deacetylase complex subunit SAP130, C-terminal domain
AAEL027494	1339.99	0.47	0.14	3.26	1.10E-03	4.51E-02	unspecified product		ATP binding	N/A	SNF2-related, N-terminal domain;Helicase, C-terminal;Helicase/SANT-
AAEL001796	272.69	0.46	0.14	3.42	6.30E-04	3.46E-02	Nuclear hormone receptor (HR78)		DNA binding;DNA-binding transcription	regulation of transcription, DNA-templated	Nuclear hormone receptor, ligand-binding domain;Zinc finger, nuclear
AAEL008938	271.95	0.45	0.13	3.36	7.71E-04	3.78E-02	unspecified product		RNA binding;nucleic acid binding	N/A	RNA recognition motif domain
AAEL001910	319.13	0.45	0.12	3.69	2.29E-04	2.13E-02	zinc finger protein		nucleic acid binding	N/A	Zinc finger C2H2-type
AAEL009080	2413.27	0.44	0.13	3.40	6.67E-04	3.50E-02	importin 7,		Ran GTPase binding	intracellular protein transport	Importin-beta, N-terminal domain;Exportin-2, central domain

AAEL014028	212.38	0.44	0.13	3.42	6.29E-04	3.46E-02	unspecified product		metallopeptidase activity;zinc ion	proteolysis	Peptidase M1, membrane alanine aminopeptidase
AAEL011147	261.55	0.43	0.13	3.34	8.45E-04	3.95E-02	unspecified product		N/A	snRNA processing	Integrator complex subunit 2
AAEL021986	268.82	0.43	0.12	3.62	2.98E-04	2.38E-02	unspecified product		RNA binding;nucleic acid binding	RNA processing	RNA recognition motif domain;SWAP/Surp
AAEL001144	298.54	0.37	0.11	3.34	8.47E-04	3.95E-02	unspecified product		protein binding	intracellular protein transport;vesicle-mediated	Citron homology (CNH) domain;9acuolar sorting protein 39/Transforming
AAEL001716	421.90	0.35	0.11	3.30	9.80E-04	4.34E-02	THO complex subunit 2 (Tho2)		N/A	mRNA export from nucleus;mRNA processing	THO complex, subunitTHOC2, C-terminal;THO complex, subunitTHOC2,
AAEL025223	412.66	0.35	0.09	3.74	1.87E-04	2.00E-02	unspecified product		protein binding	N/A	Enhancer of mRNA-decapping protein 4, WD40 repeat region
AAEL021307	1102.99	0.32	0.09	3.62	2.91E-04	2.38E-02	unspecified product		ATP binding;metalloendopeptidase	proteolysis	ATPase, AAA-type, core;Peptidase M41;AAA ATPase, AAA lid domain
AAEL001623	1580.02	-0.37	0.10	-3.63	2.87E-04	2.38E-02	proteasome activator subunit REG		N/A	N/A	Proteasome activator PA28, N-terminal domain;Proteasome activator
AAEL006169	3102.95	-0.40	0.11	-3.57	3.57E-04	2.59E-02	Lysosomal aspartic protease Precursor (EC 3.4.23.-)		aspartic-type endopeptidase activity;	proteolysis	Peptidase family A1 domain
AAEL022725	359.00	-0.40	0.12	-3.31	9.36E-04	4.22E-02	unspecified product		ATP binding;nucleotide binding;protein	N/A	Ubiquitin-conMugating enzyme E2
AAEL019972	288.02	-0.42	0.12	-3.40	6.62E-04	3.50E-02	unspecified product		N/A	cellular sphingolipid homeostasis;ceramide	ORMDL family
AAEL012276	234.89	-0.43	0.13	-3.40	6.65E-04	3.50E-02	survival motor neuron protein		RNA binding	mRNA processing	Survival motor neuron
AAEL002140	223.71	-0.44	0.14	-3.21	1.32E-03	4.98E-02	transcription initiation factor IIA (TFIIA), gamma chain		N/A	transcription initiation from RNA polymerase II	Transcription initiation factor IIA, gamma subunit, N-terminal;Transcription
AAEL014644	345.74	-0.45	0.14	-3.26	1.12E-03	4.54E-02	UDP-sugar transporter UST74c (fringe connection protein),		N/A	N/A	Sugar phosphate transporter domain
AAEL003651	595.24	-0.45	0.13	-3.44	5.92E-04	3.35E-02	unspecified product		catalytic activity	N/A	Phospholipase D/Transphosphatidylase;PLD-like domain
AAEL002933	322.34	-0.45	0.12	-3.80	1.44E-04	1.82E-02	unspecified product		N/A	N/A	Sas10/Utp3/C1D
AAEL003238	180.42	-0.46	0.14	-3.41	6.41E-04	3.46E-02	Palmitoyltransferase [Source:UniProtKB/TrEMBL;Acc:Q16L42]		acyltransferase activity;	N/A	Palmitoyltransferase, DHHC domain
AAEL007383	42961.03	-0.47	0.13	-3.66	2.52E-04	2.22E-02	secreted ferritin G subunit precursor, putative	Soma ferritin	ferric iron binding;ferroxidase activity;	cellular iron ion homeostasis;iron ion	N/A
AAEL006025	342.32	-0.47	0.12	-3.79	1.48E-04	1.85E-02	unspecified product		protein binding	N/A	Tetratricopeptide repeat 1
AAEL001250	281.97	-0.48	0.13	-3.66	2.50E-04	2.22E-02	unspecified product		N/A	N/A	Transmembrane protein 161A/B
AAEL006693	639.05	-0.48	0.15	-3.25	1.14E-03	4.60E-02	uroporphyrinogen decarboxylase		carboxy-lyase activity;lyase activity;	porphyrin-containing compound biosynthetic	Uroporphyrinogen decarboxylase (URO-D)
AAEL006676	791.82	-0.48	0.14	-3.43	6.00E-04	3.36E-02	unspecified product		N/A	N/A	FAR-17a/AIG1-like protein
AAEL008274	1245.17	-0.49	0.15	-3.31	9.36E-04	4.22E-02	9WFC domain-containing protein [Source:UniProtKB/TrEMBL;	NA	protein binding	N/A	N/A
AAEL011099	470.83	-0.49	0.15	-3.28	1.02E-03	4.41E-02	molybdopterin-binding		catalytic activity	N/A	Phosphoadenosine phosphosulphate reductase
AAEL007385	51160.74	-0.50	0.14	-3.69	2.22E-04	2.13E-02	Ferritin subunit Precursor (EC 1.16.3.1)(Ferritin heavy chain-		ferric iron binding	cellular iron ion homeostasis;iron ion transport	Ferritin/DPS protein domain
AAEL021171	239.31	-0.51	0.15	-3.28	1.03E-03	4.41E-02	unspecified product	Lamtor4 Late endosomal/lysosomal	N/A	cellular response to amino acid stimulus;	N/A
AAEL004653	380.76	-0.51	0.13	-4.05	5.02E-05	1.02E-02	elongation factor SIII p1S subunit, putative		N/A	ubiquitin-dependent protein catabolic process	SKP1 component, POZ domain
AAEL007811	190.60	-0.51	0.16	-3.26	1.13E-03	4.58E-02	short-chain dehydrogenase		protein binding	N/A	Short-chain dehydrogenase/reductase SDR;WW domain
AAEL023983	27418.67	-0.51	0.15	-3.49	4.87E-04	3.03E-02	40S ribosomal protein S4 [Source:UniProtKB/TrEMBL;Acc:		RNA binding;rRNA binding;structural	translation	Ribosomal protein S4e, central region;RNA-binding S4 domain;Ribosomal
AAEL006698	18766.17	-0.51	0.14	-3.75	1.76E-04	1.94E-02	60S ribosomal protein L31 [Source:UniProtKB/Swiss-Prot;Acc:		structural constituent of ribosome	translation	Ribosomal protein L31e
AAEL011239	197.83	-0.52	0.14	-3.61	3.03E-04	2.40E-02	short-chain dehydrogenase		N/A	N/A	Short-chain dehydrogenase/reductase SDR
AAEL008921	1817.02	-0.52	0.15	-3.38	7.29E-04	3.70E-02	myosin regulatory light chain 2 smooth muscle		calcium ion binding	N/A	EF-hand domain
AAEL015061	299.52	-0.53	0.15	-3.51	4.40E-04	2.81E-02	chaperone binding protein		N/A	N/A	CS domain;SGS domain
AAEL002372	17063.57	-0.53	0.15	-3.57	3.60E-04	2.59E-02	40S ribosomal protein S11 [Source:UniProtKB/TrEMBL;Acc:		structural constituent of ribosome	translation	Ribosomal protein S17/S11;40S ribosomal protein S11, N-terminal
AAEL001103	331.72	-0.54	0.14	-3.76	1.69E-04	1.94E-02	prefoldin, subunit, putative		unfolded protein binding	protein folding	Prefoldin beta-like
AAEL010794	386.28	-0.54	0.15	-3.53	4.16E-04	2.81E-02	prenylated rab acceptor		N/A	N/A	Prenylated rab acceptor PRA1
AAEL011087	206.05	-0.54	0.17	-3.23	1.26E-03	4.88E-02	DNA-directed RNA polymerase II		DNA binding;DNA-directed S'-3' RNA	transcription, DNA-templated	RNA polymerase, subunit H/RpbS C-terminal;RNA polymerase, RpbS, N-
AAEL008281	603.16	-0.54	0.16	-3.36	7.76E-04	3.78E-02	CRAL-TRIO domain-containing protein [Source:		N/A	N/A	CRAL-TRIO lipid binding domain
AAEL010095	679.46	-0.55	0.16	-3.46	5.40E-04	3.19E-02	unspecified product		N/A	N/A	Uncharacterised protein family, CD034/YQF4
AAEL027646	174.03	-0.55	0.16	-3.52	4.38E-04	2.81E-02	unspecified product		N/A	N/A	;RN2-binding (;TBD) domain
AAEL011665	436.82	-0.55	0.16	-3.39	7.00E-04	3.62E-02	unspecified product	NA	N/A	N/A	N/A
AAEL000847	251.57	-0.55	0.16	-3.37	7.61E-04	3.78E-02	unspecified product		N/A	N/A	C;;C motif containing zinc binding protein, eukaryotic
AAEL014198	623.25	-0.55	0.16	-3.49	4.87E-04	3.03E-02	cdp-diacylglycerol--glycerol-3-phosphate 3-		phosphotransferase activity, for other	phospholipid biosynthetic process	CDP-alcohol phosphatidyltransferase
AAEL010032	2118.74	-0.56	0.17	-3.24	1.19E-03	4.72E-02	translocon-associated protein, beta subunit precursor (trap-	SsRbeta signal sequence receptor	N/A	N/A	N/A
AAEL012417	284.24	-0.56	0.16	-3.57	3.60E-04	2.59E-02	unspecified product		acyltransferase activity	N/A	Phospholipid/glycerol acyltransferase
AAEL005694	577.84	-0.56	0.12	-4.90	9.59E-07	1.79E-03	unspecified product		catalytic activity	polyamine biosynthetic process	Insect odorant-binding protein A10/EMaculatory bulb-specific protein 3
AAEL022104	7540.81	-0.57	0.17	-3.24	1.18E-03	4.68E-02	Ribosomal protein L3 [Source:UniProtKB/TrEMBL;Acc:		structural constituent of ribosome	translation	Ribosomal protein L3
AAEL015598	123.67	-0.57	0.17	-3.36	7.84E-04	3.78E-02	unspecified product	rhythmically expressed gene 2 protein	hydrolase activity	N/A	N/A
AAEL003552	222.53	-0.57	0.15	-3.73	1.94E-04	2.00E-02	DNA-directed RNA polymerase subunit rpb6		DNA binding;DNA-directed S'-3' RNA	transcription, DNA-templated	RNA polymerase, subunit omega/K/RPB6
AAEL026904	635.89	-0.58	0.18	-3.24	1.20E-03	4.73E-02	unspecified product	60S ribosomal protein L28	N/A	N/A	N/A
AAEL012069	2017.09	-0.58	0.18	-3.22	1.30E-03	4.94E-02	glutathione peroxidase		glutathione peroxidase activity;	cellular oxidant detoxification;obsolete	Glutathione peroxidase
AAEL004404	367.66	-0.58	0.18	-3.25	1.15E-03	4.62E-02	HIG1 domain family member 2A, putative		N/A	N/A	Hypoxia induced protein, domain
AAEL008195	496.63	-0.58	0.15	-3.95	7.98E-05	1.32E-02	unspecified product		N/A	N/A	TMEM9
AAEL004860	374.28	-0.58	0.16	-3.52	4.26E-04	2.81E-02	acireductone dioxygenase		acireductone dioxygenase [iron(II)-	L-methionine salvage from	Acireductone dioxygenase ARD family
AAEL017081	946.75	-0.58	0.17	-3.41	6.39E-04	3.46E-02	unspecified product		copper chaperone activity;copper ion	copper ion transport	Cytochrome c oxidase copper chaperone
AAEL009120	156.16	-0.58	0.16	-3.59	3.37E-04	2.58E-02	cytochrome P4S0		heme binding;iron ion binding;metal	obsolete oxidation-reduction process	Cytochrome P4S0
AAEL002703	409.76	-0.58	0.17	-3.43	6.01E-04	3.36E-02	pyridoxamine S'-phosphate oxidase		FMN binding;obsolete cofactor	obsolete oxidation-reduction process;	Pyridoxamine S'-phosphate oxidase, putative;Pyridoxine S'-phosphate
AAEL013689	1182.54	-0.59	0.16	-3.75	1.76E-04	1.94E-02	neuronal calcium sensor, putative		calcium ion binding	N/A	EF-hand domain
AAEL007185	281.18	-0.59	0.17	-3.55	3.81E-04	2.67E-02	unspecified product		N/A	vesicle-mediated transport	Trafficking protein particle complex subunit
AAEL009995	359.66	-0.60	0.15	-3.89	9.83E-05	1.50E-02	transmembrane protein, putative		N/A	cell redox homeostasis	Thioredoxin domain;[NiFe]-hydrogenase maturation factor HyaE
AAEL009431	198.92	-0.61	0.17	-3.48	4.92E-04	3.04E-02	mitochondrial ribosomal protein, L10, putative		N/A	ribosome biogenesis	Ribosomal protein L10P
AAEL005508	778.02	-0.61	0.16	-3.75	1.74E-04	1.94E-02	NADH-ubiquinone oxidoreductase 24 kda subunit	ND-24 NADH dehydrogenase	2 iron, 2 sulfur cluster binding;iron-	obsolete oxidation-reduction process	N/A
AAEL001831	205.42	-0.61	0.16	-3.80	1.42E-04	1.82E-02	DNA-directed RNA polymerase II		DNA-directed S'-3' RNA polymerase	mRNA cleavage;transcription, DNA-templated	Zinc finger, TFIIS-type;DNA-directed RNA polymerase, M/1SkDa subunit
AAEL009101	2268.82	-0.61	0.16	-3.75	1.75E-04	1.94E-02	Eukaryotic translation initiation factor 3 subunit F (eIF3f)		protein binding;translation initiation	cytoplasmic translational initiation;formation of	-AB1/MPN/MO934 metalloenzyme domain;Rpn11/EIF3F, C-terminal
AAEL007472	136.93	-0.61	0.16	-3.69	2.25E-04	2.13E-02	unspecified product		N/A	N/A	UbiB domain
AAEL003582	21643.64	-0.61	0.15	-4.05	5.02E-05	1.02E-02	40S ribosomal protein S13 [Source:UniProtKB/TrEMBL;Acc:		structural constituent of ribosome	translation	Ribosomal protein S1S;Ribosomal protein S13/S1S, N-terminal
AAEL000951	5819.21	-0.61	0.17	-3.62	2.93E-04	2.38E-02	elongation factor 1-beta2		translation elongation factor activity	translation;translational elongation	Translation elongation factor EF1B, beta/delta subunit, guanine nucleotide
AAEL023382	195.53	-0.62	0.19	-3.25	1.17E-03	4.68E-02	unspecified product		N/A	N/A	Uncharacterised protein family UPF0390
AAEL025437	380.82	-0.62	0.16	-3.94	8.20E-05	1.32E-02	unspecified product		oxidoreductase activity	obsolete oxidation-reduction process	FAD dependent oxidoreductase

AAEL004884	598.63	-0.62	0.15	-4.11	3.97E-05	9.26E-03	hemomucin		strictosidine synthase activity	biosynthetic process	Strictosidine synthase, conserved region
AAEL006859	156.15	-0.62	0.19	-3.26	1.11E-03	4.54E-02	Myb-interacting protein, putative		N/A	N/A	Protein LIN37
AAEL011587	19276.28	-0.62	0.15	-4.16	3.17E-05	8.40E-03	60S ribosomal protein L27, putative		structural constituent of ribosome	translation	KOW;Ribosomal protein L27e
AAEL010299	11125.13	-0.62	0.17	-3.70	2.17E-04	2.13E-02	40S ribosomal protein S12 [Source:UniProtKB/TrEMBL;Acc:		structural constituent of ribosome	translation	Ribosomal protein L7Ae/L30e/S12e/Gadd4S
AAEL012674	264.51	-0.63	0.17	-3.68	2.31E-04	2.13E-02	d-amino acid oxidase		D-amino-acid oxidase activity;FAD	D-amino acid metabolic process;obsolete	FAD dependent oxidoreductase
AAEL006115	1497.74	-0.63	0.13	-4.78	1.80E-06	2.59E-03	Eukaryotic translation initiation factor 3 subunit K (eIF3k)(eIF-3		RNA binding;ribosome binding;	cytoplasmic translational initiation;formation of	CSN8/PSMD8/EIF3K
AAEL002337	169.62	-0.63	0.19	-3.32	9.06E-04	4.17E-02	prefoldin, subunit, putative		unfolded protein binding	protein folding	Prefoldin beta-like
AAEL024536	26962.03	-0.63	0.15	-4.28	1.89E-05	8.40E-03	Ribosomal protein L19 [Source:UniProtKB/TrEMBL;Acc:		RNA binding;structural constituent of	translation	Ribosomal protein L19/L19e
AAEL005939	100.06	-0.63	0.19	-3.31	9.18E-04	4.18E-02	mpv17 protein		N/A	N/A	Mpv17/PMP22
AAEL005817	21917.06	-0.64	0.15	-4.16	3.18E-05	8.40E-03	60S ribosomal protein L26 [Source:UniProtKB/TrEMBL;Acc:		structural constituent of ribosome	translation	KOW;Ribosomal protein L26/L24, eukaryotic/archaeal
AAEL010168	26545.98	-0.64	0.18	-3.62	2.92E-04	2.38E-02	40S ribosomal protein S2 [Source:UniProtKB/TrEMBL;Acc:		RNA binding;structural constituent of	translation	Ribosomal protein SS, N-terminal;Ribosomal protein SS, C-terminal
AAEL020238	19645.60	-0.64	0.18	-3.46	5.37E-04	3.19E-02	unspecified product		structural constituent of ribosome	ribosomal small subunit assembly;translation	Ribosomal protein S2;40S ribosomal protein SA, C-terminal domain
AAEL002534	5842.02	-0.64	0.17	-3.87	1.10E-04	1.58E-02	60S ribosomal protein L10 [Source:UniProtKB/TrEMBL;Acc:		structural constituent of ribosome	translation	Ribosomal protein L10e/L16
AAEL028043	5655.57	-0.64	0.17	-3.74	1.83E-04	1.98E-02	60S ribosomal protein L28, putative [Source:		structural constituent of ribosome	translation	Ribosomal L28e/Mak16
AAEL008719	142.61	-0.64	0.19	-3.38	7.24E-04	3.69E-02	Sm protein G, putative		RNA binding	RNA splicing;mRNA processing;mRNA	LSM domain, eukaryotic/archaea-type
AAEL004043	935.68	-0.65	0.17	-3.70	2.13E-04	2.12E-02	unspecified product		N-acetyltransferase activity;	N/A	FR47-like;Domain of unknown function DUFS64S
AAEL005901	11709.96	-0.65	0.17	-3.81	1.39E-04	1.82E-02	40S ribosomal protein S3a [Source:UniProtKB/Swiss-Prot;Acc:		structural constituent of ribosome	translation	Ribosomal protein S3Ae
AAEL019789	2720.07	-0.65	0.19	-3.41	6.58E-04	3.50E-02	40S ribosomal protein S3a [Source:UniProtKB/Swiss-Prot;Acc:		structural constituent of ribosome	translation	Ribosomal protein S3Ae
AAEL021595	78.24	-0.65	0.19	-3.37	7.53E-04	3.76E-02	unspecified product	ElpS Elongator complex protein S	N/A	tRNA wobble uridine modification	N/A
AAEL008481	23001.73	-0.65	0.16	-4.08	4.46E-05	9.68E-03	60S ribosomal protein L18 [Source:UniProtKB/Swiss-Prot;Acc:		structural constituent of ribosome	translation	Ribosomal protein L18e/L1SP
AAEL012078	563.64	-0.66	0.16	-4.06	5.01E-05	1.02E-02	Gamma-glutamylcyclotransferase [Source:		gamma-glutamylcyclotransferase	glutathione catabolic process	Glutathione-specific gamma-glutamylcyclotransferase
AAEL013221	21266.73	-0.66	0.17	-3.86	1.14E-04	1.61E-02	60S ribosomal protein L10a		RNA binding;structural constituent of	translation	Ribosomal protein L1/ribosomal biogenesis protein
AAEL004430	158.23	-0.66	0.18	-3.58	3.46E-04	2.59E-02	mitochondrial ribosomal protein, L30, putative		structural constituent of ribosome	translation	Ribosomal protein L30, ferredoxin-like fold domain
AAEL011447	25731.09	-0.66	0.17	-3.99	6.59E-05	1.16E-02	60S ribosomal protein L14 [Source:UniProtKB/TrEMBL;Acc:		RNA binding;structural constituent of	translation	Ribosomal protein L14e domain
AAEL000987	29505.34	-0.67	0.17	-3.89	1.01E-04	1.52E-02	60S ribosomal protein L8		structural constituent of ribosome	translation	Ribosomal Proteins L2, RNA binding domain;Ribosomal protein L2, C-
AAEL005097	21845.92	-0.68	0.19	-3.56	3.74E-04	2.64E-02	cold induced protein (BnC24A), putative		structural constituent of ribosome	translation	Ribosomal protein L13e
AAEL006779	85.46	-0.68	0.21	-3.27	1.06E-03	4.49E-02	cytochrome c oxidase assembly protein cox11		copper ion binding	N/A	Cytochrome c oxidase assembly protein CtaG/Cox11
AAEL014562	15821.51	-0.68	0.16	-4.15	3.38E-05	8.40E-03	60S ribosomal protein L12		structural constituent of ribosome	translation	Ribosomal protein L11, C-terminal;Ribosomal protein L11, N-terminal
AAEL010756	16107.16	-0.68	0.21	-3.21	1.33E-03	4.99E-02	40S ribosomal protein S19 [Source:UniProtKB/TrEMBL;Acc:		structural constituent of ribosome	translation	Ribosomal protein S19e
AAEL013272	13434.05	-0.69	0.19	-3.52	4.25E-04	2.81E-02	60S ribosomal protein L37a		structural constituent of ribosome	translation	Ribosomal protein L37ae
AAEL006566	121.79	-0.69	0.18	-3.76	1.73E-04	1.94E-02	suppressor of ty		zinc ion binding	positive regulation of DNA-templated	Spt4/RpoE2 zinc finger
AAEL011832	335.04	-0.69	0.19	-3.58	3.49E-04	2.59E-02	unspecified product		RNA binding;nucleic acid binding	N/A	K Homology domain, type 1
AAEL008192	16153.58	-0.69	0.18	-3.80	1.44E-04	1.82E-02	40S ribosomal protein S3 [Source:UniProtKB/TrEMBL;Acc:		RNA binding;structural constituent of	translation	Ribosomal protein S3, C-terminal;K Homology domain, type 2
AAEL009653	14105.12	-0.69	0.22	-3.22	1.29E-03	4.91E-02	40S ribosomal protein S30 [Source:UniProtKB/TrEMBL;Acc:		protein binding;structural constituent of	translation	Ribosomal protein S30
AAEL013625	30009.63	-0.70	0.18	-3.78	1.58E-04	1.88E-02	40S ribosomal protein SS [Source:UniProtKB/TrEMBL;Acc:		RNA binding;structural constituent of	translation	Ribosomal protein S7 domain
AAEL007699	24058.19	-0.70	0.17	-4.18	2.87E-05	8.40E-03	60S ribosomal protein L9 [Source:UniProtKB/TrEMBL;Acc:		rRNA binding;structural constituent of	translation	Ribosomal protein L6, alpha-beta domain
AAEL000820	713.43	-0.70	0.22	-3.23	1.25E-03	4.86E-02	dimethylaniline monooxygenase		N,N-dimethylaniline monooxygenase	obsolete oxidation-reduction process	Flavin monooxygenase-like
AAEL000677	126.42	-0.71	0.22	-3.22	1.29E-03	4.91E-02	DNA-directed RNA polymerase subunit rpb8		N/A	transcription by RNA polymerase II;	RNA polymerase, Rpb8
AAEL023681	13894.61	-0.71	0.19	-3.79	1.51E-04	1.86E-02	unspecified product	60S ribosomal protein L34	N/A	N/A	N/A
AAEL001759	18438.39	-0.71	0.20	-3.62	2.90E-04	2.38E-02	40S ribosomal protein S9 [Source:UniProtKB/TrEMBL;Acc:		RNA binding;rRNA binding;structural	translation	Ribosomal protein S4/S9, N-terminal;RNA-binding S4 domain
AAEL013554	512.13	-0.71	0.14	-4.99	5.95E-07	1.39E-03	cytochrome P4S0		heme binding;iron ion binding;metal	obsolete oxidation-reduction process	Cytochrome P4S0
AAEL005266	13753.66	-0.71	0.18	-3.88	1.03E-04	1.53E-02	40S ribosomal protein S14 [Source:UniProtKB/Swiss-Prot;Acc:		structural constituent of ribosome	translation	Ribosomal protein S11
AAEL002183	588.17	-0.71	0.22	-3.22	1.29E-03	4.91E-02	oligosaccharyl transferase, subunit, putative		dolichyl-diphosphooligosaccharide-	protein glycosylation	DAD/Ost2
AAEL007715	16729.56	-0.71	0.19	-3.72	1.97E-04	2.00E-02	60S ribosomal protein L21 [Source:UniProtKB/TrEMBL;Acc:		structural constituent of ribosome	translation	Ribosomal protein L21e
AAEL008329	16819.90	-0.71	0.17	-4.21	2.56E-05	8.40E-03	60S ribosomal protein L24 [Source:UniProtKB/TrEMBL;Acc:		N/A	N/A	Ribosomal protein L24e-related
AAEL006557	289.74	-0.71	0.17	-4.14	3.51E-05	8.40E-03	unspecified product		DNA binding	N/A	PDCDS-like
AAEL002832	24069.01	-0.71	0.22	-3.24	1.21E-03	4.73E-02	40S ribosomal protein S26 [Source:UniProtKB/TrEMBL;Acc:		structural constituent of ribosome	translation	Ribosomal protein S26e
AAEL003396	4107.22	-0.72	0.19	-3.72	1.97E-04	2.00E-02	60S ribosomal protein L32 [Source:UniProtKB/TrEMBL;Acc:		structural constituent of ribosome	translation	Ribosomal protein L32e
AAEL002047	14784.62	-0.72	0.20	-3.64	2.78E-04	2.38E-02	40S ribosomal protein S10 [Source:UniProtKB/TrEMBL;Acc:		N/A	N/A	Plectin/S10, N-terminal
AAEL001838	292.86	-0.72	0.19	-3.78	1.60E-04	1.88E-02	unspecified product		unfolded protein binding	protein folding	Prefoldin beta-like
AAEL008188	31671.03	-0.72	0.16	-4.41	1.05E-05	6.07E-03	60S ribosomal protein L6 [Source:UniProtKB/TrEMBL;Acc:		structural constituent of ribosome	translation	60S ribosomal protein L6E
AAEL012818	95.83	-0.72	0.22	-3.22	1.27E-03	4.91E-02	Protein SYS1 homolog [Source:UniProtKB/TrEMBL;Acc:		N/A	protein transport	Integral membrane protein SYS1-related
AAEL004175	2672.50	-0.72	0.17	-4.21	2.54E-05	8.40E-03	40S ribosomal protein S17 [Source:UniProtKB/TrEMBL;Acc:		structural constituent of ribosome	translation	Ribosomal protein S17e
AAEL012440	6002.26	-0.73	0.17	-4.21	2.59E-05	8.40E-03	sodium-bile acid cotransporter		N/A	N/A	Bile acid:sodium symporter/arsenical resistance protein Acr3
AAEL005220	12909.51	-0.73	0.20	-3.57	3.56E-04	2.59E-02	60S ribosomal protein L30 [Source:UniProtKB/TrEMBL;Acc:		RNA binding;structural constituent of	N/A	Ribosomal protein L7Ae/L30e/S12e/Gadd4S
AAEL005914	128.45	-0.73	0.20	-3.60	3.17E-04	2.49E-02	unspecified product		N/A	N/A	N/A
AAEL000823	21366.43	-0.73	0.18	-4.03	5.54E-05	1.06E-02	60S ribosomal protein L3SA, putative [Source:		structural constituent of ribosome	translation	Ribosomal protein L3SA
AAEL013533	6175.30	-0.73	0.22	-3.27	1.07E-03	4.49E-02	unspecified product		N/A	N/A	N/A
AAEL013069	25104.76	-0.73	0.19	-3.82	1.36E-04	1.82E-02	guanine nucleotide-binding protein subunit beta-like protein		kinase activity;protein binding	phosphorylation	WD40 repeat
AAEL000467	136.80	-0.73	0.22	-3.37	7.53E-04	3.76E-02	mitochondrial ribosomal protein, L49, putative		structural constituent of ribosome	translation	Ribosomal protein L49/IMG2
AAEL015006	20272.06	-0.73	0.21	-3.46	5.38E-04	3.19E-02	60S ribosomal protein L23 (L17A)(AeRpL17A)		structural constituent of ribosome	translation	Ribosomal protein L14P
AAEL014583	20107.68	-0.73	0.18	-4.00	6.46E-05	1.16E-02	60S acidic ribosomal protein P2 [Source:UniProtKB/TrEMBL;		structural constituent of ribosome	translational elongation	N/A
AAEL003921	101.94	-0.74	0.18	-4.20	2.68E-05	8.40E-03	ubiquitin, putative		protein binding	N/A	Ubiquitin-like domain
AAEL011471	23370.16	-0.74	0.22	-3.34	8.24E-04	3.89E-02	60S ribosomal protein L17 [Source:UniProtKB/Swiss-Prot;Acc:		structural constituent of ribosome	translation	Ribosomal protein L22/L17
AAEL009151	20249.59	-0.74	0.16	-4.49	7.20E-06	5.87E-03	40S ribosomal protein S1Sa		structural constituent of ribosome	translation	Ribosomal protein S8
AAEL004335	803.07	-0.74	0.22	-3.40	6.66E-04	3.50E-02	secreted ferritin G subunit precursor, putative		ferric iron binding;ferroxidase activity;	cellular iron ion homeostasis;iron ion	N/A
AAEL009747	20241.13	-0.74	0.18	-4.20	2.69E-05	8.40E-03	40S ribosomal protein S18 [Source:UniProtKB/TrEMBL;Acc:		RNA binding;nucleic acid binding;	translation	Ribosomal protein S13
AAEL006511	18707.74	-0.74	0.16	-4.54	5.51E-06	5.87E-03	ubiquitin		protein binding;structural constituent of	translation	Ubiquitin-like domain;Ribosomal protein L40e
AAEL006908	223.82	-0.75	0.17	-4.44	8.81E-06	5.87E-03	unspecified product		N/A	N/A	PITH domain

AAEL020749	21837.61	-0.75	0.20	-3.65	2.64E-04	2.29E-02	60S ribosomal protein L1S/L27 [Source:UniProtKB/TrEMBL;	structural constituent of ribosome	translation	Ribosomal protein L18e/L1SP
AAEL012686	28455.91	-0.75	0.19	-4.02	5.79E-05	1.08E-02	40S ribosomal protein S23 [Source:UniProtKB/TrEMBL;Acc:	structural constituent of ribosome	translation	Ribosomal protein S12/S23
AAEL005722	33811.97	-0.75	0.17	-4.45	8.56E-06	5.87E-03	60S ribosomal protein L7a	N/A	ribosome biogenesis	Ribosomal protein L7Ae/L30e/S12e/Gadd4S
AAEL024434	12139.77	-0.75	0.18	-4.09	4.25E-05	9.44E-03	60S ribosomal protein L13a (Fragment) [Source:	structural constituent of ribosome	translation	Ribosomal protein L13
AAEL005629	14956.47	-0.76	0.19	-4.00	6.22E-05	1.14E-02	60S ribosomal protein L3S, putative	structural constituent of ribosome	translation	Ribosomal protein L29/L3S
AAEL014292	15274.73	-0.76	0.20	-3.80	1.42E-04	1.82E-02	40S ribosomal protein S24 [Source:UniProtKB/TrEMBL;Acc:	structural constituent of ribosome	translation	Ribosomal protein S24e
AAEL028141	270.58	-0.76	0.22	-3.52	4.40E-04	2.81E-02	unspecified product	N/A	N/A	N/A
AAEL001588	2297.51	-0.76	0.22	-3.45	5.59E-04	3.24E-02	glutamate carboxypeptidase	dipeptidase activity;hydrolase activity;	proteolysis	Peptidase M20;Peptidase M20, dimerisation domain
AAEL007771	15627.30	-0.77	0.22	-3.46	5.34E-04	3.19E-02	60S ribosomal protein L22 [Source:UniProtKB/TrEMBL;Acc:	structural constituent of ribosome	translation	Ribosomal protein L22e
AAEL011656	20363.06	-0.77	0.20	-3.87	1.09E-04	1.58E-02	40S ribosomal protein S1S [Source:UniProtKB/TrEMBL;Acc:	RNA binding;structural constituent of	translation	Ribosomal protein S19/S1S
AAEL020352	648.66	-0.77	0.22	-3.57	3.60E-04	2.59E-02	unspecified product	N/A	N/A	N/A
AAEL005451	17680.58	-0.77	0.17	-4.42	9.81E-06	6.07E-03	60S ribosomal protein L38 [Source:UniProtKB/Swiss-Prot;Acc:	structural constituent of ribosome	translation	Ribosomal protein L38e
AAEL006270	88.35	-0.77	0.22	-3.52	4.31E-04	2.81E-02	Sugar transporter SWEET [Source:UniProtKB/TrEMBL;Acc:	N/A	carbohydrate transport	SWEET sugar transporter
AAEL012944	19147.96	-0.77	0.18	-4.20	2.68E-05	8.40E-03	60S ribosomal protein L11 [Source:UniProtKB/TrEMBL;Acc:	structural constituent of ribosome	translation	Ribosomal protein LS, N-terminal;Ribosomal protein LS, C-terminal
AAEL021083	22769.48	-0.78	0.18	-4.22	2.46E-05	8.40E-03	40S ribosomal protein S2S [Source:UniProtKB/TrEMBL;Acc:	N/A	N/A	Ribosomal protein S2S
AAEL006860	15231.45	-0.78	0.24	-3.27	1.08E-03	4.49E-02	40S ribosomal protein S28 [Source:UniProtKB/TrEMBL;Acc:	structural constituent of ribosome	translation	Ribosomal protein S28e
AAEL004151	11592.31	-0.78	0.18	-4.36	1.31E-05	6.81E-03	60S ribosomal protein L29 [Source:UniProtKB/TrEMBL;Acc:	structural constituent of ribosome	translation	Ribosomal protein L29e
AAEL003530	5354.09	-0.78	0.23	-3.43	6.13E-04	3.41E-02	acidic ribosomal protein P1, putative	structural constituent of ribosome	translation;translational elongation	N/A
AAEL011217	84.84	-0.78	0.23	-3.46	5.50E-04	3.23E-02	unspecified product	N/A	N/A	PQ-loop repeat
AAEL007243	136.01	-0.79	0.19	-4.14	3.46E-05	8.40E-03	valacyclovir hydrolase	N/A	N/A	Alpha/beta hydrolase fold-1
AAEL022286	23817.10	-0.79	0.19	-4.17	3.06E-05	8.40E-03	40S ribosomal protein S27 [Source:UniProtKB/TrEMBL;Acc:	metal ion binding;structural constituent	translation	Ribosomal protein S27e
AAEL003427	19326.01	-0.80	0.19	-4.14	3.48E-05	8.40E-03	40S ribosomal protein S16 [Source:UniProtKB/Swiss-Prot;Acc:	structural constituent of ribosome	translation	Ribosomal protein S9
AAEL024747	135.84	-0.80	0.21	-3.84	1.24E-04	1.73E-02	unspecified product	N/A	N/A	Electron transfer flavoprotein, alpha subunit, C-terminal
AAEL003942	8971.19	-0.80	0.20	-4.03	5.57E-05	1.06E-02	60S ribosomal protein L44 L41, putative	structural constituent of ribosome	translation	Ribosomal protein L44e
AAEL010582	591.87	-0.81	0.25	-3.27	1.09E-03	4.49E-02	glutathione transferase	protein binding;transferase activity	N/A	Glutathione S-transferase, C-terminal;Glutathione S-transferase, N-terminal
AAEL007387	392.48	-0.82	0.25	-3.28	1.04E-03	4.43E-02	unspecified product	protein binding	N/A	Histidine phosphatase superfamily, clade-1;Ubiquitin-associated domain;
AAEL008553	122.26	-0.87	0.22	-3.94	8.04E-05	1.32E-02	unspecified product	N/A	N/A	Cytochrome c oxidase assembly protein CO;14
AAEL000720	41.67	-0.88	0.25	-3.45	5.63E-04	3.24E-02	mediator of RNA polymerase II transcription subunit 11	transcription coregulator activity	regulation of transcription by RNA polymerase	Mediator complex, subunit Med11
AAEL004804	74.98	-0.88	0.21	-4.15	3.35E-05	8.40E-03	hexaprenyldihydroxybenzoate methyltransferase	2-polyprenyl-6-methoxy-1,4-	methylation;ubiquinone biosynthetic process	N/A
AAEL002874	46.80	-0.90	0.27	-3.39	7.05E-04	3.62E-02	apolipoprotein a binding protein	NADH; epimerase activity;isomerase	N/A	YMeF N-terminal domain
AAEL007947	115.99	-0.93	0.27	-3.42	6.35E-04	3.46E-02	glutathione transferase	protein binding	N/A	Glutathione S-transferase, C-terminal;Glutathione S-transferase, N-terminal
AAEL020284	71.78	-0.94	0.29	-3.29	1.01E-03	4.40E-02	unspecified product	N/A	N/A	N/A
AAEL004465	63.74	-0.96	0.23	-4.15	3.32E-05	8.40E-03	unspecified product	ATPase activator activity;chaperone	positive regulation of ATP-dependent activity;	Dna- domain;Co-chaperone HscB, C-terminal oligomerisation domain
AAEL010338	23808.73	-0.98	0.28	-3.55	3.90E-04	2.71E-02	unspecified product	N/A	N/A	Protein TsetseEP
AAEL004684	94.47	-1.00	0.23	-4.40	1.11E-05	6.07E-03	unspecified product	lipid transfer activity	intermembrane lipid transfer	Glycolipid transfer protein domain
AAEL013045	51.73	-1.07	0.31	-3.47	5.27E-04	3.19E-02	exosome complex exonuclease RRP41, putative	N/A	N/A	Exoribonuclease, phosphorolytic domain 1
AAEL006134	64.98	-1.09	0.26	-4.25	2.18E-05	8.40E-03	unspecified product	N/A	N/A	Coiled-coil domain-containing protein 134
AAEL026174	116.91	-1.16	0.35	-3.33	8.70E-04	4.02E-02	unspecified product	N/A	intracellular cholesterol transport	MD-2-related lipid-recognition domain
AAEL006903	110.79	-1.19	0.32	-3.69	2.21E-04	2.13E-02	trypsin	hydrolase activity;peptidase activity;	proteolysis	Serine proteases, trypsin domain
AAEL021848	29.86	-1.21	0.34	-3.56	3.67E-04	2.62E-02	unspecified product	N/A	N/A	N/A
AAEL012852	4042.75	-1.23	0.37	-3.30	9.52E-04	4.25E-02	trypsin	hydrolase activity;peptidase activity;	proteolysis	Serine proteases, trypsin domain
AAEL025157	21.52	-1.58	0.40	-3.92	8.68E-05	1.37E-02	unspecified product	N/A	N/A	N/A
AAEL013283	7.07	-5.82	1.47	-3.95	7.71E-05	1.31E-02	serine-type endopeptidase	hydrolase activity;peptidase activity;	proteolysis	Serine proteases, trypsin domain

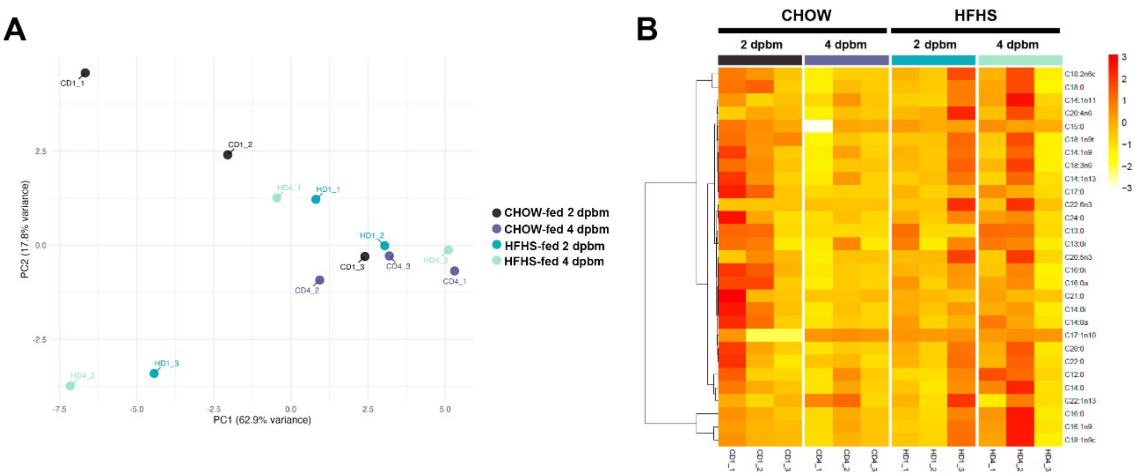

Supplemental Figure 1. Fatty acid storage in Mosquito Fat Bodies. *Aedes aegypti* mosquitoes were fed on AG129 mice subjected to either CHOW or HFHS diets. Following, fat body-enriched abdominal carcasses were dissected at 2 and 4 dpbm, and homogenized. Fatty acid content was analyzed by GC-MS/MS. (A) Principal Component Analysis and (B) heatmap representation. A total of 3 independent mice feeding were used for each condition (CHOW or HFHS). Each replicate was comprised of a batch of 15 pooled mosquitoes, which had been fed on an independent mouse.

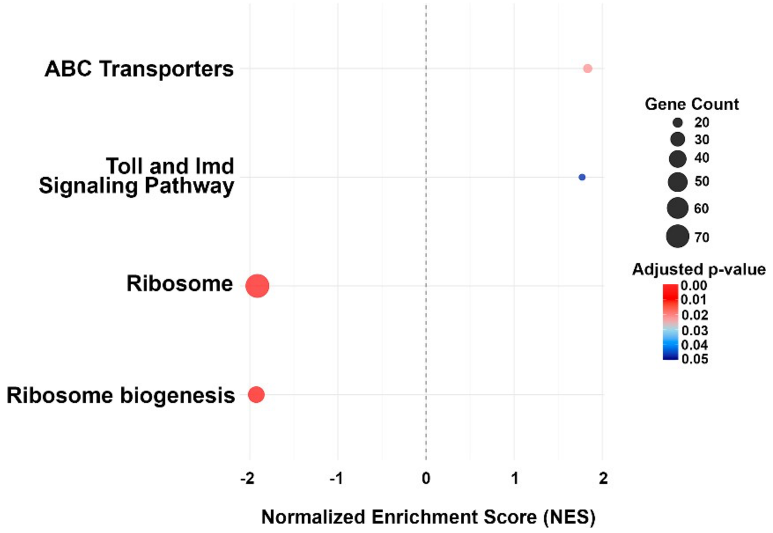

Supplemental Figure 2. KEGG Pathway Enrichment in the Midgut of Naive *A. aegypti* Mosquitoes Fed on CHOW or HFHS Diets 4 days post-blood meal. Enriched KEGG pathways were identified using GSEA. Statistical significance for gene expression analysis was determined using DESeq2, and pathway enrichment was assessed using GSEA with KEGG database annotations. The Normalized Enrichment Score (NES) of differentially modulated pathways is plotted on the X-axis, with positive NES values representing upregulated pathways and negative NES values representing suppressed pathways in CHOW-fed mosquitoes. Bubble size represents the gene count (number of "core enrichment" genes that significantly contribute to the enrichment score), while color indicates adjusted p-value. A dashed vertical line at NES = 0 separates activated from suppressed pathways. A total of 4 independent blood feedings were used per condition (CHOW or HFHS), where each replicate consisted of a pool of 15 mosquito midguts.

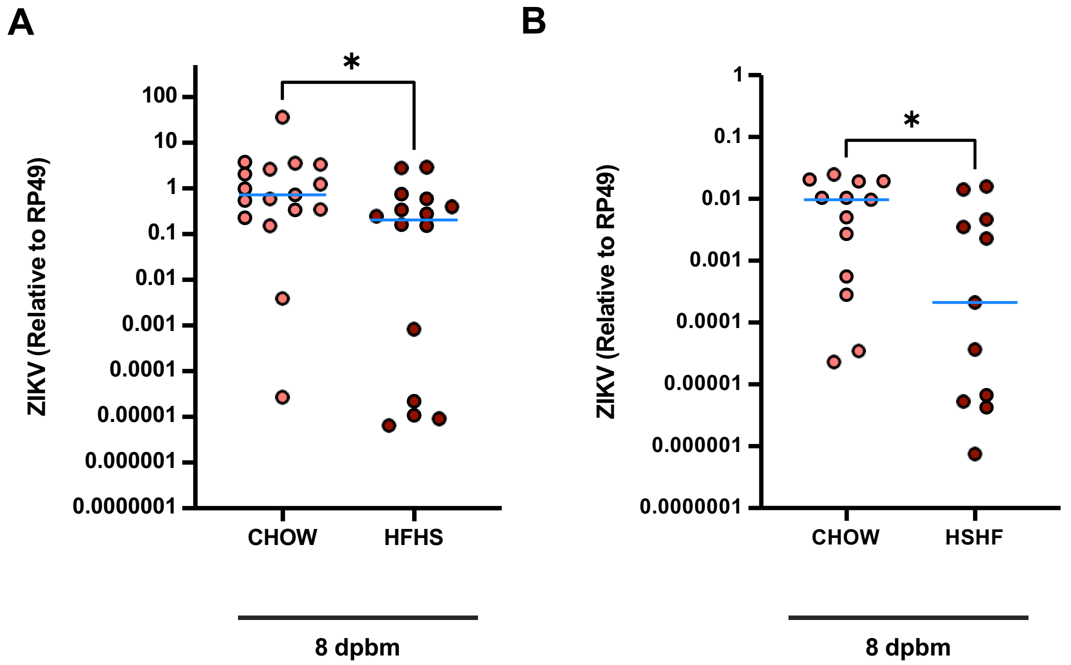

Supplemental Figure 3. ZIKV Infection in Mosquito Midguts 8 Days Post-Infection. *A. aegypti* mosquitoes were fed with the blood of AG129 mice subjected to either CHOW or HFHS diets supplemented with ZIKV. (A) Midgut and (B) fat body-enriched abdominal carcass were dissected at 8 dpbm, and infection levels were quantified by qRT-PCR. Individual ZIKV RNA levels per mosquito (log scale) was plotted. ZIKV statistical analysis was performed using the Mann-Whitney unpaired test *: P<0.05. A total of (A) CHOW: 17, HFHS: 16 and (B) CHOW: 13, HFHS: 11 individual mosquitoes were analyzed.

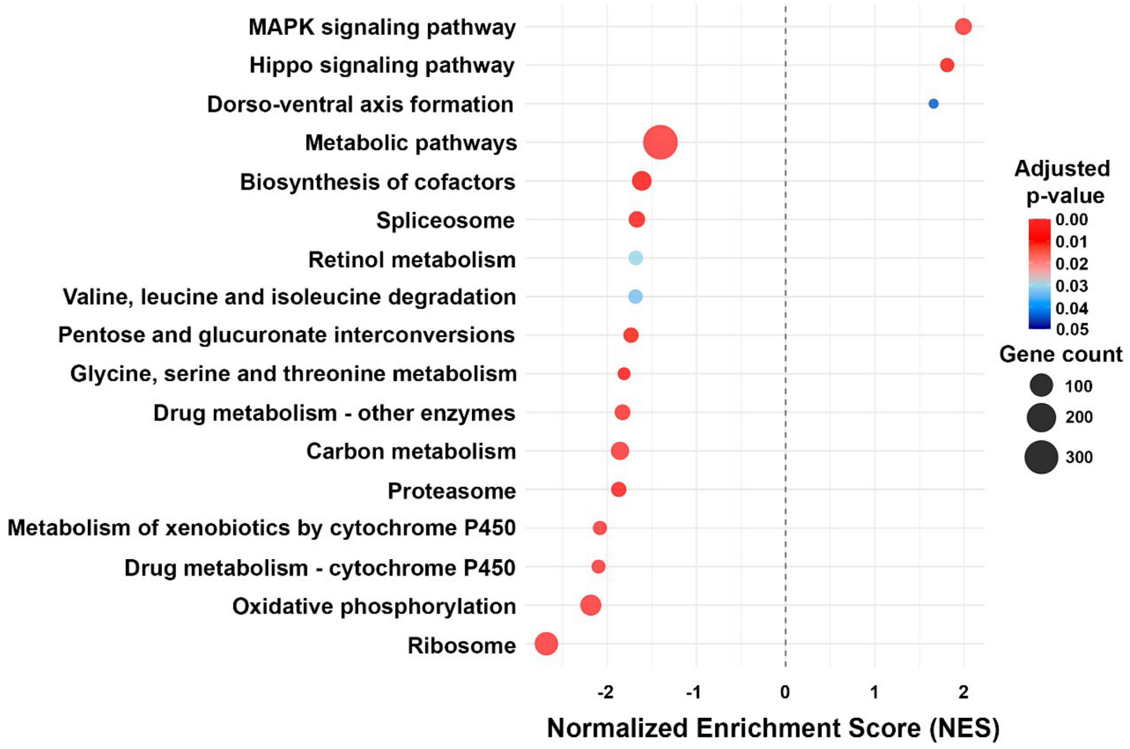

Supplemental Figure 4. KEGG Pathway Enrichment in the Midgut of ZIKV-Infected *A. aegypti* Mosquitoes Fed on CHOW or HFHS Diets 4 days Post Infection. Enriched KEGG pathways were identified using GSEA. Statistical significance for gene expression analysis was determined using DESeq2, and pathway enrichment was assessed using GSEA with KEGG database annotations. The Normalized Enrichment Score (NES) of differentially modulated pathways is plotted on the X-axis, with positive NES values representing upregulated pathways and negative NES values representing suppressed pathways in CHOW-fed mosquitoes. Bubble size represents the gene count (number of "core enrichment" genes that significantly contribute to the enrichment score), while color indicates adjusted p-value. A dashed vertical line at NES = 0 separates activated from suppressed pathways. A total of 4 independent blood feedings were used per condition (CHOW or HFHS), where each replicate consisted of a pool of 15 mosquito midguts.
